# Effect of direct-fed microbials on culturable gut microbiotas in broiler chickens: a meta-analysis of controlled trials

**DOI:** 10.5713/ajas.18.0009

**Published:** 2018-05-31

**Authors:** Chhaiden Heak, Peerapol Sukon, Pairat Sornplang

**Affiliations:** 1 Faculty of Veterinary Medicine, Khon Kaen University, Khon Kaen 40002, Thailand; 2 Research Group for Animal Health Technology, Khon Kaen University, Khon Kaen 40002, Thailand

**Keywords:** Direct-fed Microbials, Probiotics, Broiler Chicken, Gut Microbiota, Systematic Review, Meta-analysis

## Abstract

**Objective:**

This meta-analysis was conducted to evaluate the overall effect of direct-fed microbial (DFM) or probiotic supplementation on the log concentrations of culturable gut microbiota in broiler chickens.

**Methods:**

Relevant studies were collected from PubMed, SCOPUS, Poultry Science Journal, and Google Scholar. The studies included controlled trials using DFM supplementation in broiler chickens and reporting log concentrations of the culturable gut microbiota. The overall effect of DFM supplementation was determined using standardized mean difference (SMD) with a random-effects model. Subgroups were analyzed to identify pre-specified characteristics possibly associated with the heterogeneity of the results. Risk of bias and publication bias were assessed.

**Results:**

Eighteen taxa of the culturable gut microbiota were identified from 42 studies. The overall effect of DFM supplementation on the log concentrations of all 18 taxa did not differ significantly from the controls (SMD = −0.06, 95% confidence interval [−0.16, 0.04], p = 0.228, I^2^ = 85%, n = 699 comparisons), but the 18 taxa could be further classified into three categories by the direction of the effect size: taxa whose log concentrations did not differ significantly from the controls (category 1), taxa whose log concentrations increased significantly with DFM supplementation (category 2), and taxa whose log concentrations decreased significantly with DFM supplementation (category 3). Category 1 comprised nine taxa, including total bacterial counts. Category 2 comprised four taxa: *Bacillus*, *Bifidobacterium*, *Clostridium butyricum*, and *Lactobacillus*. Category 3 comprised five taxa: *Clostridium perfringens*, coliforms, *Escherichia coli*, *Enterococcus*, and *Salmonella*. Some characteristics identified by the subgroup analysis were associated with result heterogeneity. Most studies, however, were present with unclear risk of bias. Publication bias was also identified.

**Conclusion:**

DFM supplementation increased the concentrations of some beneficial bacteria (e.g. *Bifidobacterium* and *Lactobacillus*) and decreased those of some detrimental bacteria (e.g. *Clostridium perfringens* and *Salmonella*) in the guts of broiler chickens.

## INTRODUCTION

Direct-fed microbials (DFMs), or probiotics, are live microorganisms (mostly bacteria or yeast) that may confer health benefits on the host. DFM supplementation has been extensively studied in broiler chickens to replace or reduce the use of antibiotics as growth promoters. The mechanisms of DFMs for health benefits on the host are not fully understood and may involve several beneficial mechanisms [1]. Modification of the gut microbiota may be the most important mechanism, because the gut microbiota, a very complex microbial community, plays an important role in maintaining homeostasis and the physiology of the host, increasing health and productivity [2]. In addition to its direct contribution to host health, the gut microbiota of broiler chickens may also have indirect negative impacts on human and environmental health, because some microbes (as a part of gut microbiotas), such as *Campylobacter* spp. commonly shed and excreted in the feces, are harmful human pathogens [3]. These microbes may contaminate the food chain or environment and threaten human health.

Modification of the gut microbiota by increasing beneficial bacteria and decreasing harmful pathogens in the gastrointestinal tract is therefore the primary goal of DFM application in broiler chickens. DFM supplementation in broiler chickens can substantially alter (either increase or decrease) the populations of particular bacterial species in the gastrointestinal tract [4]. Recent advances in molecular techniques, especially next-generation sequencing, have led to a better understanding of the very diverse and complex communities of gut microbiotas in broiler chickens [5,6], but most of the currently available evidence for the effect of DFMs on gut microbiotas have come from studies using culture-based techniques for determining concentrations of the gut bacteria of interest. We therefore conducted this systematic review and meta-analysis to determine the overall effect of DFM supplementation on the log concentrations of culturable gut microbiotas in broiler chickens and to identify pre-defined characteristics (factors) that may be associated with the heterogeneity of the results.

## MATERIALS AND METHODS

### Review protocol

The protocol for this study was developed using SYRCLE’s protocol format [7] to minimize bias. This protocol is available in the supplementary information. Deviations from the protocol are identified in the relevant sections.

### Information sources and search strategy

We collected relevant citations from PubMed, Scopus, Poultry Science Journal, and Google Scholar databases using the keywords: DFM, probiotic, microbiota, normal flora, and chicken. An example of a search algorithm for PubMed was: ((((“probiotics”[MeSH Terms] OR “probiotics”[All Fields] OR “probiotic”[All Fields]) OR (direct[All Fields] AND fed[All Fields] AND microbial[All Fields])) OR (“microbiota”[MeSH Terms] OR “microbiota”[All Fields])) OR (normal[All Fields] AND (“Flora”[Journal] OR “flora”[All Fields]))) AND (“chickens” [MeSH Terms] OR “chickens”[All Fields] OR “chicken”[All Fields]). This study was limited to English publications. The last search was completed on 23 May 2016.

### Eligibility criteria and study selection

Two independent reviewers assessed study eligibility with two screening steps. The reviewers first assessed the titles and abstracts of retrieved citations. Full-text articles were then assessed if the titles and abstracts passed the first screening step. The criteria for study selection were: i) randomized or non-randomized controlled trials with DFM supplementation as an intervention in broiler chickens, and ii) only studies reporting concentrations of the culturable gut microbiota either in the gastrointestinal tract or in the excreta. Studies were excluded with the following criteria: i) reviews, reports, errata, and duplicated articles, ii) not about broiler chickens, iii) trials with no control group and *in vitro* or *in ovo* studies, iv) treatments using non-viable DFMs, treatments other than DFMs (e.g. prebiotics, synbiotics, medicinal plants, or enzymes), or a combination of DFMs with other products, v) incomplete reporting of outcome data, and vi) trials with disease challenges or antimicrobial products. A PRISMA flow chart for systematic reviews was used to summarize the study selection.

### Study characteristics and data extraction

Two independent reviewers extracted the available data from the included studies. The following data were collected for each study: i) bibliographic data (author names, publication year), ii) study design (number of animals, number of treatments and replicates, number of birds per pen or block), iii) characteristics of experimental chickens (broiler breed, sex, group sample size), iv) characteristics of intervention (DFM species and strains, product classification, administration dose, application frequency and route, duration of treatment), v) outcome measures (concentrations of culturable microbiota in the crop, small and large intestines, cecum, and excreta). Selection of the pre-defined characteristics or factors was based on information from the previous studies [8,9]. Some characteristics were used for subgroup analysis to explore heterogeneity of the results. Any inconsistencies between the reviewers were resolved by discussion.

### Assessment of risk of bias

Two independent reviewers assessed the risk of bias for the included studies using SYRCLE’s RoB tool [10]. The reported details of each study were evaluated for 10 categorical domains of bias: *Selection bias* (domains 1–3), *Performance bias* (domains 4 and 5), *Detection bias* (domains 6 and 7), *Attrition bias* (domain 8), *Reporting bias* (domain 9), and *Others* (domain 10) [10]. Each domain was assigned to one of three categories: “yes” indicated a low risk of bias, “no” indicated a high risk of bias, and “?” indicated an unclear risk of bias.

### Outcome measure and statistical analysis

The outcome measure was the standardized mean difference (SMD) of the log concentrations of the culturable gut microbiota isolated from the chicken gastrointestinal tract (crop, small and large intestines, and cecum) or the excreta. Comprehensive Meta-Analysis version 3 (Biostat, Englewood, NJ, USA) was used for all analyses. Universal Desktop Ruler version 3 was used to convert graphically reported outcomes. Standard errors or standard errors of the mean were converted to standard deviations. As a pre-specified model of choice, a random-effects model was used for all analyses to obtain an overall or individual effect size with its 95% confidence interval (CI). Differences were considered significant at p<0.05. The heterogeneity of the results from the studies was assessed using Cochran’s Q and qualified by I^2^. The percentage of I^2^ <25%, 50% to 75%, and >75% indicated low, moderate, and high heterogeneity, respectively [11]. We identified 18 bacterial taxa of the culturable gut microbiota available for meta-analysis, so we classified these taxa into three categories based on the direction of the effect size to simplify the interpretation: taxa whose log concentrations did not differ significantly from the controls (category 1), taxa whose log concentrations increased significantly with DFM supplementation (category 2), and taxa whose log concentrations decreased significantly with DFM supplementation (category 3). This classification was not pre-specified in the study protocol.

Subgroup analysis was pre-specified for eight characteristics: broiler breed, sex, DFM product, DFM species, sampling organ, application frequency, application route, and application duration. Each characteristic was categorized into various subgroups (based on available information from the studies): broiler breed with eight subgroups (Arbor Acres, Cobb, Cuban EB24, Lingnan Yellow, Ross, Sasso X44, Tsukuba jidori, and unknown), sex with three subgroups (male, both male and female, and unknown), DFM product with two subgroups (commercial and non-commercial), DFM species with three subgroups (single, multiple, and unknown), sampling organ with seven subgroups (crop, duodenum, ileum, cecum, colon, excreta, and unknown), application route with four subgroups (feed, gavage, water, and both feed and water), application frequency with two subgroups (daily and once), and application duration with five subgroups (≤7, >7 and ≤14, >14 and ≤21, >21 and ≤14, and >42 days of age). The subgroups were analyzed only if each subgroup contained at least four studies (or four outcome comparisons) [12] with at least two subgroups for each characteristic. Subgroups were analyzed only for *Lactobacillus* (chosen based on the large number of outcome comparisons and as a representative of beneficial bacteria) and for a combination of coliforms and *Escherichia coli* (*E. coli*) (chosen based on the large number of outcome comparisons and as representatives of detrimental bacteria) to reduce the number of subgroup comparisons and to simplify subgroup interpretation. This restriction was not pre-specified in the protocol but was based on available information after the primary meta-analysis. A sensitivity analysis was conducted to assess the robustness of the results because of decisions made during the systematic review; we evaluated the influences of model selection (random versus fixed). Publication bias was assessed visually using a funnel plot and was tested formally by Egger’s test, with p<0.10 indicating the presence of publication bias [13]. When publication bias was found, Duval and Tweedie’s trim and fill methods were used to estimate the effect size of possibly missing publications [14].

## RESULTS

### Search results and study selection

**Figure 1:**
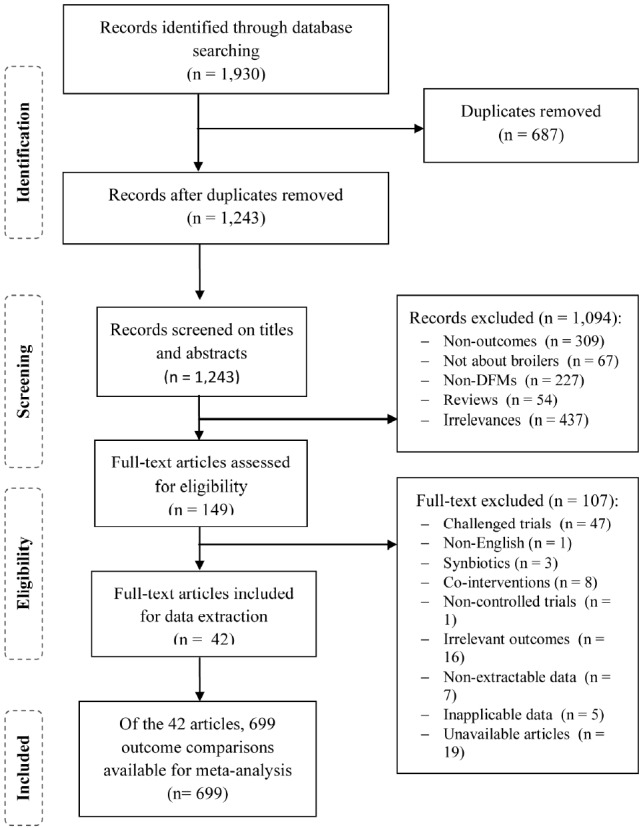
Flow diagram of study selection.

A total of 1930 citations published between 1950 and 2016 were identified. Of these, 687 were identified as duplicates, and 1,094 did not pass the first screening step (Figure 1), so 149 full-text articles were assessed for study eligibility. Of these, 42 full-text articles (with a total of 699 outcome comparisons) were included for data extraction and meta-analysis. The references of all included studies are presented in the supplementary information (Supplementary Table S5).

### Characteristics of included studies

The detailed characteristics of the 42 studies are provided in Supplementary Table S1 in the supplementary information. The 42 studies with 699 outcome comparisons included 18 culturable bacterial microbiota taxa (Table 1) and were published between 1996 and 2016. Most of the studies (36/42) were defined as randomized controlled trials. Ross was a broiler breed frequently used in the trials (22/42), and two studies reported unknown or unspecified breeds. Male chicks were frequently used in the trials (24/42), and an unknown or unspecified sex was occasionally found (11/42). Commercial DFM products were used in 17 studies. A single DFM species was used in 28 studies. The concentration of microbials used in treatments ranged from 10^5^ to 10^12^ cfu/kg, and the microbials were normally mixed in feed (38/42) with daily supplementation (41/42).

### Primary analysis

**Figure 2:**
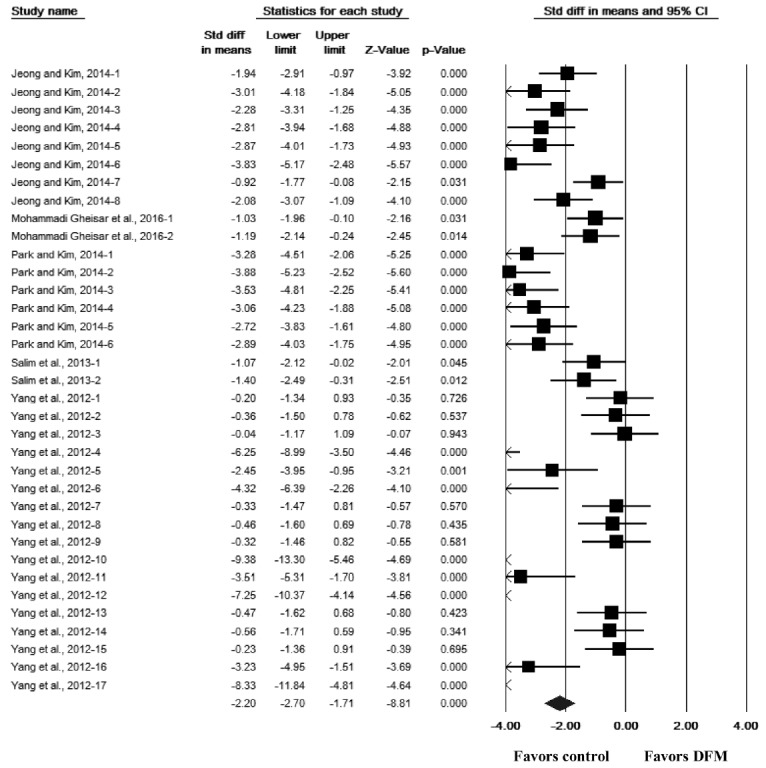
Forest plot for the effect of direct-fed microbial (DFM) supplementation on the log concentrations of *Salmonella* in the gut of broiler chickens. For each comparison, a black box and its horizontal line represent the point estimate and its 95% confidence interval (CI) of a standardized mean difference (SMD), respectively. The box or its horizontal line that crosses the middle vertical line (the line of 0.00) indicates a non-significant result. The size of the box was proportional to the weight used in meta analysis; the bigger the box, the more weight. The diamond at the bottom of the forest plot represents the overall SMD or the summary effect from all comparisons. The center of the diamond and its lateral tips represent the point estimate and its 95% CI of the overall SMD, respectively.

The effect of DFM supplementation on the log concentrations of the 18 bacterial taxa (based on the culture media) are also presented in Table 1. The overall effect of DFM supplementation did not differ significantly between the 18 taxa and the controls (SMD = −0.06, 95% CI [−0.16, 0.04], p = 0.228, n = 699 comparisons) and was highly heterogeneous among the studies (Q = 4585.78, p<0.001, I^2^ = 85%). Category 1 of the 18 taxa comprised nine taxa (aerobes, anaerobes, *Bacteroides*, *Clostridium coccoides*, *Enterobacteriaceae*, Gram-positive cocci, mesophilic bacteria, *Streptococcus*, and total bacterial counts); the overall effect of DFM supplementation did not differ significantly from the controls (SMD = −0.12, 95% CI [−0.35, 0.11], p = 0.298, n = 130 comparisons) and were highly heterogeneous (Q = 592.62, p<0.001, I^2^ = 78%). Category 2 comprised four taxa (*Bacillus*, *Bifidobacterium*, *Clostridium butyricum* [*C. butyricum*], and *Lactobacillus*); the overall effect of DFM supplementation were associated with a significant increase in the log concentrations of bacterial counts compared with the controls (SMD = 1.07, 95% CI [0.92, 1.22], p<0.001, n = 279 comparisons) and were highly heterogeneous (Q = 1,283.64, p<0.001, I^2^ = 78%). Category 3 comprised five taxa (*Clostridium perfringens* [*C. perfringens*], coliforms, *E. coli*, *Enterococcus*, and *Salmonella*); the overall effect of DFM supplementation was associated with a significant decrease in the log concentrations of bacterial counts compared with the controls (SMD = −1.26, 95% CI [−1.42, −1.10], p<0.001, n = 290 comparisons) and were highly heterogeneous (Q = 1,526.73, p<0.001, I^2^ = 81%). As an example, a forest plot for the effects of DFM supplementation on the log concentrations of Salmonella is shown in Figure 2. Forest plots for the remaining bacterial taxa are provided in the supplementary information (Supplementary Figures S1–S5).

### Subgroup analysis

The results of the subgroup analysis of eight characteristics are presented in Table 2 (for *Lactobacillus*) and 3 (for coliforms and *E. coli*). For *Lactobacillus*, three characteristics differed significantly among the subgroups: broiler breed, microbial species, and application duration (p<0.001 for each characteristic) (Table 2). For breed, the log concentration of *Lactobacillus* after DFM supplementation increased the most in Cuban EB24 (SMD = 2.13, 95% CI [0.85, 3.42], p<0.001, n = 6 comparisons), followed by Ross, Arbor Acres, Cobb, and Lingnan Yellow. For DFM species, the effect of DFM supplementation on the log concentrations of *Lactobacillus* was greatest for an unknown (unspecified) DFM species (SMD = 2.38, 95% CI [0.02, 4.73], p = 0.048, n = 5 comparisons), followed by single and multiple DFM species. For application duration, the effect of DFM supplementation was greatest when applied between 21 and 42 days of age (SMD = 1.23, 95% CI [0.98, 1.49], p<0.001, n = 95 comparisons).

For the combination of coliforms and *E. coli*, three characteristics differed significantly among the subgroups: broiler breed, sampling organ, and application duration (p<0.001 for each characteristic) (Table 3). For broiler breed, the log concentrations of coliforms and *E. coli* decreased the most in an unknown (unspecified) breed (SMD = −5.60, 95% CI [−7.87, −3.33], p<0.001, n = 12 comparisons), followed by Ross, Lingnan Yellow and Arbor Acres. For sampling organ, the log concentrations of coliforms and *E. coli* decreased the most in the colon (SMD = −3.76, 95% CI [−5.26, −2.27], p<0.001, n = 14 comparisons), followed by the cecum and ileum. For application duration, the log concentrations of coliforms and *E. coli* decreased the most when the supplementation was applied between 21 and 42 days of age (n = 98 comparisons, SMD = −1.14, 95% CI [−1.39, −0.88], p<0.001).

### Sensitivity analysis

The results of the sensitivity analysis for comparing a random-effects model (the model of choice for this study) with a fixed-effects model are presented in Table 4. The random-effects model found larger effect sizes than the fixed-effects model for all three categories of taxa, but the directions of the effect sizes and the results of the statistical tests were similar for all three categories. The SMDs (the magnitudes of the effect sizes) for the random-and fixed-effects models were −0.12 and −0.08, respectively, for category 1 (taxa whose log concentrations did not differ significantly from the controls, n = 130 comparisons); 1.07 and 0.77, respectively, for category 2 (taxa whose log concentrations increased significantly with DFM supplementation, n = 279 comparisons); and −1.26 and −0.89, respectively, for category 3 (taxa whose log concentrations decreased significantly with DFM supplementation, n = 290 comparisons).

### Assessment of risk of bias

**Figure 3:**
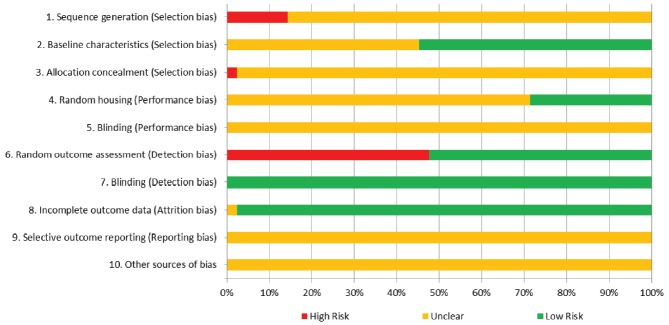
Risk of bias presented as the percentage of the 42 studies. All or almost all articles had unclear risk of bias for domains 1, 3, 5, 9, and 10, indicating insufficient information from the articles to determine the risk status for those domains. Blinding for detection bias (domain 7) was considered low risk for all articles because the log concentration of the bacterial count was not a subjective outcome.

Overall results from the assessment of risk of bias for the 42 studies for each domain are presented in Figure 3 (results from the assessment for each article are available in Supplementary Table S2). For sequence generation (domain 1), 36 of the 42 articles (86%) had unclear risks of bias, and six articles (14%) had high risks of bias. For baseline characteristics (domain 2), 23 of the 42 articles (55%) had low risks of bias, and 19 articles (45%) had unclear risks of bias. For allocation concealment (domain 3), 36 of the 42 articles (86%) had unclear risks of bias. For random housing (domain 4), 30 of the 42 articles (71%) had unclear risks of bias, and 12 articles (29%) had low risks of bias. For blinding of the caregivers (domain 5), all 42 articles had unclear risks of bias. For random-outcome assessment (domain 6), 20 of the 42 articles (48%) had high risks of bias, and 22 articles (52%) had low risks of bias. For blinding of outcome assessors (domain 7), all 42 articles had low risks of bias. For incomplete outcome data (domain 8), 41 of the 42 articles (98%) had low risks of bias, and one article (2%) had an unclear risk of bias. For selective outcome reporting (domain 9) and other sources of bias (domain 10), all 42 articles had unclear risks of bias.

### Assessment of publication bias

**Figure 4:**
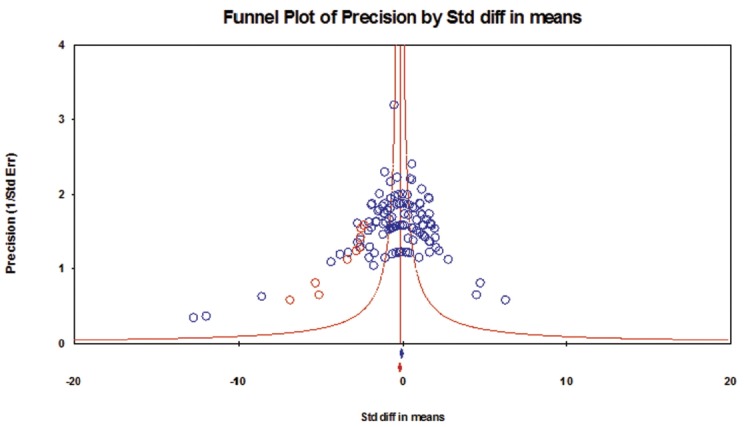
Funnel plot for bacteria in category 1 (taxa whose log concentrations did not differ significantly from the controls). Y-axis represents the study precision (the inverse of standard error) and x-axis shows the standardized mean difference (SMD). A blue circle represents each comparison (n = 130) of the included studies. Distribution of the blue circles was approximate symmetry around a red vertical line (the line corresponding to the overall SMD), indicating that publication bias was not apparent. Only nine missing comparisons (red circles) were imputed as suggested by trim and fill analysis. A blue and a red marker at the bottom of the funnel plot represent the overall SMD before and after missing comparisons were imputed, respectively.

**Figure 5:**
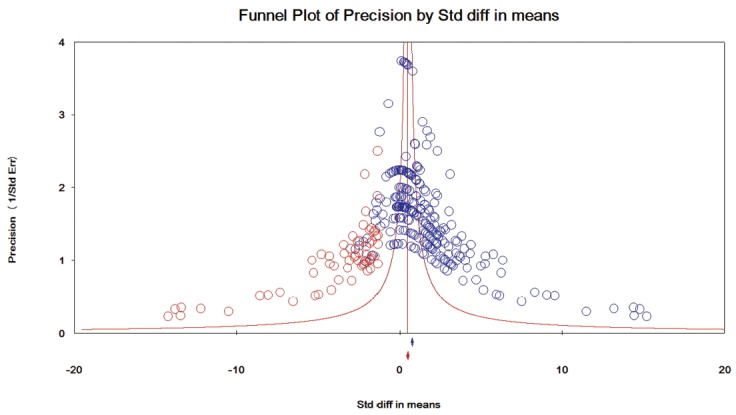
Funnel plot for bacteria in category 2 (taxa whose log concentrations increased significantly with DFM supplementation). Y-axis represents the study precision (the inverse of standard error) and x-axis shows the standardized mean difference (SMD). A blue circle represents each comparison (n = 279) of the included studies. Distribution of the blue circles was clearly asymmetric around a red vertical line (the line corresponding to the overall SMD), indicating that publication bias was apparent. As many as 62 missing comparisons (red circles) were imputed on the left hand side of the plot as suggested by trim and fill analysis. A blue and a red marker at the bottom of the funnel plot represent the overall SMD before and after missing comparisons were imputed, respectively.

**Figure 6:**
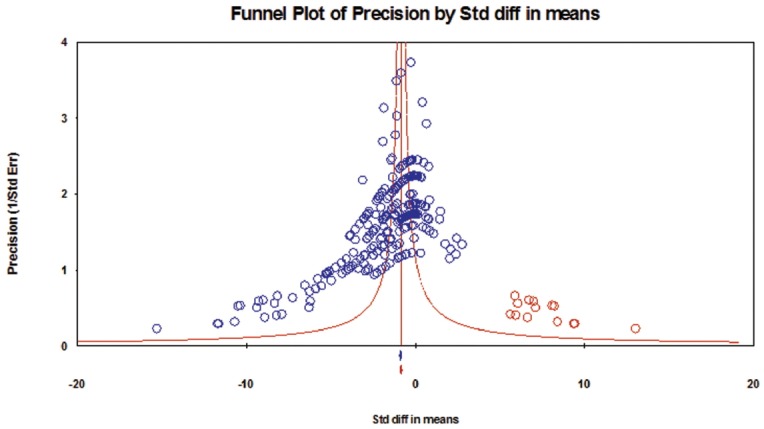
Funnel plot for bacteria in category 3 (taxa whose log concentrations decreased significantly with DFM supplementation). Y-axis represents the study precision (the inverse of standard error) and x-axis shows the standardized mean difference (SMD). A blue circle represents each comparison (n = 290) of the included studies. Distribution of the blue circles was clearly asymmetric around a red vertical line (the line corresponding to the overall SMD), indicating that publication bias was apparent. Fourteen missing comparisons (red circles) were imputed on the right hand side of the plot as suggested by trim and fill analysis. A blue and a red marker at the bottom of the funnel plot represent the overall SMD before and after missing comparisons were imputed, respectively.

The results of the assessment of publication bias for each category of taxa are presented in Figures 4–6. In category 1 (taxa whose log concentrations did not differ significantly from the controls, n = 130 comparisons), publication bias was not apparent in the funnel plot (Figure 4), and Egger’s test was not significant (p = 0.182). In category 2 (taxa whose log concentrations increased significantly with DFM supplementation, n = 279 comparisons), the funnel plot clearly indicated publication bias (Figure 5), and Egger’s test was significant (p<0.001). Duval and Tweedie’s trim and fill methods indicated 62 missing comparisons for this category, which adjusted SMD to 0.54 (95% CI, 0.37 to 0.71) from the original SMD of 1.07 (95% CI, 0.92 to 1.22). In category 3 (taxa whose log concentrations decreased significantly with DFM supplementation, n = 290 comparisons), the funnel plot clearly indicated publication bias (Figure 6), and Egger’s test for publication bias was significant (p<0.001).

## DISCUSSION

Our meta-analysis suggests that DFM supplementation can confer health benefits to broiler chickens by increasing the concentrations of some beneficial bacteria and decreasing those of some detrimental bacteria. DFM supplementation did not significantly alter the overall log concentrations of culturable gut microbiotas, including total bacterial counts, but it significantly increased the log concentrations of four taxa (*Bacillus*, *Bifidobacterium*, *C. butyricum*, and *Lactobacillus*) and significantly decreased those of five taxa (*C. perfringens*, coliforms, *E. coli*, *Enterococcus*, and *Salmonella*).

In the group whose log concentrations increased significantly, *Bifidobacterium* and *Lactobacillus*, genera predominantly found in the gastrointestinal tracts of animals, including chickens, are beneficial to the host and generally regarded as safe [15]. Increasing their concentrations would therefore confer health benefits to the host, which has led to extensive investigation of their probiotic potentials in a variety of animal species [16,17]. Twenty-three of the 42 studies used *Lactobacillus* in DFM supplementation, indicating its popularity for application in broiler chickens. *Lactobacillus*, a Gram-positive, facultative anaerobic or microaerophilic, rod-shaped, non-spore-forming bacterium belonging to the phylum Firmicutes, has several beneficial effects on the host, e.g. producing digestive enzymes, helping to breakdown bile salts, helping the synthesis of vitamins B and K, and enhancing innate and acquired immunity [18]. Our results of increased concentrations of *Lactobacillus* were consistent with those in other studies, such as in pigs [19], dogs [20], and mice [21]. Whether the increased concentrations of *Lactobacillus* were due to DFM supplementation or to indigenous bacteria, however, remains unclear because of study limitations identifying bacterial origins.

Only 5 of the 42 studies used *Bifidobacterium* in DFM supplementation, indicating its lower popularity for application in broiler chickens. The lower popularity may be due to the lower abundance of *Bifidobacterium* than *Lactobacillus* in the gastrointestinal tract of chickens [2]. *Bifidobacterium*, a Gram-positive, non-motile, often branched anaerobic bacterium belonging to the phylum Actinobacteria, however, is very important in humans because it is found predominantly in infants [22]. A significant change in bifidobacterial number or composition is associated with several gastrointestinal disorders in humans, such as irritable bowel syndrome and inflammatory bowel disease [23]; many *Bifidobacterium* species and strains have therefore been identified and used as probiotics in humans [24].

Sixteen of the 42 studies used *Bacillus* in DFM supplementation, indicating moderate application in broiler chickens. *Bacillus* is a Gram-positive, rod-shaped, endospore-forming, aerobic or facultatively anaerobic bacterium belonging to the phylum Firmicutes. *Bacillus* is of great interest as a probiotic candidate in broiler chickens because it can produce durable spores that can germinate in extreme environments such as gastrointestinal tracts and during food processing. *Bacillus* can also produce various beneficial substances such as antimicrobial compounds, enzymes, and vitamins [25]. Only two species of *Bacillus* were used in the 15 studies; 13 used *Bacillus subtilis* (*B. subtilis*) and two used *Bacillus amyloliquefaciens*, indicating the importance of *B. subtilis* as a DFM product in broiler chickens. Some *Bacillus* species, though, are pathogenic, such as *Bacillus anthracis* (the causative agent of anthrax) and *Bacillus cereus* (a causative agent of food poisoning). The pros and cons of the *Bacillus* genus have raised safety concerns for its use in probiotics, so individual *Bacillus* strains or species must be evaluated for safety for each application [25]. Information for *Bacillus* was available in our study at the genus level, so the health benefits from increasing the log concentrations of *Bacillus* remained unclear.

Several mechanisms have been proposed for the alteration or modulation of gut microbiota by DFMs or probiotics. DFMs can secrete antimicrobial agents or other metabolic agents that suppress the growth of other microbes or compete for binding sites on the gut mucosa [26], balancing beneficial and detrimental microbes in the gut. This balance or homeostasis is very important for host health. Unbalanced gut microbiotas (dysbiosis or dysbacteriosis) [27] are associated with several gastrointestinal diseases, such as necrotic enteritis in chickens [28]. Many factors other than DFM can determine or alter the gut microbiota in chickens, e.g. dietary components, antibiotic growth promoters, prebiotics [29], feeding patterns [30], and litter [31]. Detailed contents of these factors are beyond the scope of this discussion. A review by Stanley et al [2] provide excellent information.

DFM supplementation has been strongly associated with increasing beneficial bacteria and decreasing detrimental bacteria in the gut of broiler chickens, but our results indicated high heterogeneity among studies, even after subgroup analysis, i.e. the effects of DFM supplementation varied from one study to another.

The results from the subgroup analysis indicated that the effectiveness of DFM supplementation may differ among the subgroups of some characteristics. The subgroup differences were found in three characteristics (broiler breed, microbial species, and application duration) for the log concentrations of *Lactobacillus* (Table 2) and three characteristics (broiler breed, sampling organ, and application duration) for those of coliforms and *E. coli* (Table 3). For the log concentrations of *Lactobacillus*, Cuban EB24 and Ross were associated with greater effect size. However, the estimated effect size for Cuban EB24 was not precise because a CI of the estimate was substantially wide with the very small number of studies (n = 6). For the log concentrations of coliforms and *E. coli*, Ross and unknown breeds were associated with greater effect size. We found that 14 studies reported unknown breeds for the subgroup comparisons; as a result, this caused a difficulty in interpretation due to the limited information about the breeds. The discrepancy or heterogeneity on the log concentrations of *Lactobacillus* and a combination of coliforms and *E. coli* among breeds of chickens can be explained that the genetic background (chicken type and breed) has been considered as a factor influencing the gut microbiota composition [32]. In turn, the gut microbiota from each breed or even individual animals may interact differently with DFM supplementation. This interaction along with other influencing factors may result in the different response from each breed or individual animals. Surprisingly, we found that increase in the log concentrations of *Lactobacillus* was greater in the application of single DFM species than that of multiple DFM species. These results were opposite to our expectation because many studies showed that an application of multiple DFM or multiple probiotic species would get better results [33,34]. These unexpected results could be explained that most trials in broiler chickens used *Lactobacillus*-based DFM with known and higher concentrations than multiple species-based DFM. The log concentrations of *Lactobacillus* detected in the samples came from a combination of the indigenous bacteria and the bacteria of DFM supplementation. Application duration between 21 and 42 days of age was associated with the greatest increase in the log concentrations of *Lactobacillus* and with the greatest decrease in those of coliforms and *E. coli*. These findings indicated that DFM supplementation would help to reduce a risk of detrimental bacterial contaminations in commercial broiler products. The age of chickens and the time point at which DFM supplementation is administered are well-known factors for its effectiveness [35]. Additional subgroup analysis was performed on DFM supplementation at a genus level (results are present in Supplementary Table S3–S4 of the supplementary data). A significant subgroup difference was observed for both the log concentrations of *Lactobacillus* and those of coliforms and *E. coli*. This indicated that genus, species, or even strains of DFM may be associated with the effectiveness. In addition, many other factors (including environment factors) may affect the gut microbiota in broiler chickens. These factors included housing, feed, litter, hygiene, climate and geographical location [36]. It is possible that these factors may also influence the effectiveness of DFM supplementation. The residual heterogeneity within each subgroup for each character after the subgroup analysis, however, was still high (Tables 3, 4), indicating that other (unknown) factors were responsible for the heterogeneity. Heterogeneous effects of DFMs or probiotics have been identified in several meta-analyses, e.g. the effect of probiotic supplementation on growth performance in broiler chickens [37]. This variability may account for the low acceptance of DFM or probiotic supplementation as a routine practice in the poultry industry, compared with other interventions such as antibiotics.

Our study has several limitations that should be taken into account when interpreting the results. First, the measured outcome is specific to the log concentration of the culturable gut microbiota. The majority of the gut microbiota cannot be cultured, so the results of this study cannot represent the entire microbiota but just some species of interest of culturable microbiota that have been reported. The use of techniques not based on culturability, especially next-generation sequencing, will allows us to better understand the abundance and diversity of the gut microbiota. This information, however, is currently too limited in trials of DFM supplementation in broiler chickens to synthesize the data. Second, the heterogeneity of outcomes is high even after accounting for subgroup analysis. Results are thus quite variable among studies, and characteristics other than our pre-specified characteristics in the subgroup analysis were responsible for the remaining heterogeneity. Third, most studies did not have clear risks of bias for most of the domains assessed, indicating that insufficient information was available for judging the bias. Our conclusions may change if new information indicates clear risks of bias. Fourth, publication bias was clear in the outcome for categories 2 (taxa whose log concentrations increased significantly with DFM supplementation) and 3 (taxa whose log concentrations decreased significantly with DFM supplementation) but not 1 (taxa whose log concentrations did not differ significantly from the controls). Publication bias arises from many sources and can distort the effect size of the outcome under study [38].

In conclusion, this systematic review and meta-analysis found that DFM supplementation modulated the gut microbiotas of broiler chickens by increasing the log concentrations of beneficial bacteria (*Bacillus*, *Bifidobacterium*, *C. butyricum*, and *Lactobacillus*) and decreasing those of detrimental bacteria (*C. perfringens*, coliforms, *E. coli*, *Enterococcus*, and *Salmonella*), suggesting health benefits of DFM supplementation for broiler chickens. This conclusion, however, was based on highly heterogeneous results among the studies, unclear risks of bias for reporting quality assessment, and publication bias of the available data in reporting bacterial taxa with statistically significant tests.

## Figures and Tables

**Table 1:** Primary analysis of the effect of DFM supplementation on the log concentrations of gut microbiotas in broiler chickens

Bacterial taxon	n1)	Effect size			Heterogeneity		
	
		SMD	[95% CI]	p value	Q	p value	I^2^
Category 12)							
Aerobes	19	−0.02	[−0.67, 0.64]	0.962	97.85	<0.001	82
Anaerobes	66	−0.34	[−0.70, 0.02]	0.066	367.94	<0.001	82
*Bacteroides*	4	−0.35	[−0.93, 0.23]	0.236	2.20	0.532	00
*Clostridium coccoides*	2	−0.03	[−0.77, 0.71]	0.941	0.05	0.830	00
*Enterobacteriaceae*	11	0.06	[−0.53, 0.65]	0.839	30.23	<0.001	67
Gram+ve cocci	9	0.49	[−0.29, 1.27]	0.217	31.40	<0.001	75
Mesophilic bacteria	8	0.64	[−0.07, 1.35]	0.078	18.94	0.008	63
*Streptococcus*	4	−0.82	[−1.66, 0.01]	0.054	4.76	0.190	37
Total bacterial counts	7	−0.10	[−1.02, 0.82]	0.831	17.41	0.008	66
Overall	130	−0.12	[−0.35, 0.11]	0.298	592.62	<0.001	78
Category 23)							
*Bacillus*	9	0.54	[0.01, 1.06]	0.045	40.55	<0.001	80
*Bifidobacterium*	58	1.32	[1.01, 1.64]	<0.001	226.63	<0.001	75
*Clostridium butyricum*	7	5.11	[2.84, 7.39]	<0.001	47.64	<0.001	87
*Lactobacillus*	205	0.96	[0.78, 1.13]	<0.001	902.68	<0.001	77
Overall	279	1.07	[0.92, 1.22]	<0.001	1,283.64	<0.001	78
Category 34)							
*C. perfringens*	58	−1.60	[−2.00, −1.21]	<0.001	341.14	<0.001	83
*Coliforms*	106	−0.81	[−1.05, −0.56]	<0.001	538.61	<0.001	81
*Escherichia coli*	83	−1.20	[−1.45, −0.94]	<0.001	304.50	<0.001	73
*Enterococcus*	8	−1.21	[−2.04, −0.38]	0.004	33.00	<0.001	79
*Salmonella*	35	−2.20	[−2.70, −1.71]	<0.001	183.56	<0.001	81
Overall	290	−1.26	[−1.42, −1.10]	<0.001	1,526.73	<0.001	81
Overall effect of DFM	699	−0.06	[−0.16, 0.04]	0.228	4,585.78	<0.001	85

DFM, direct-fed microbial; SMD, standardized mean difference for the log concentrations; CI, confidence interval.

1) n, number of outcome comparisons.

2) Category 1, bacteria whose log concentrations did not differ significantly from the controls.

3) Category 2, bacteria whose log concentrations increased significantly with DFM supplementation.

4) Category 3, bacteria whose log concentrations decreased significantly with DFM supplementation.

**Table 2:** Subgroup analysis of the effect of DFM supplementation on the log concentrations of Lactobacillus in broiler chickens

Characteristics and subgroups	Effect size				Heterogeneity			p value for subgroup difference
	
	n1)	SMD	[95% CI]	p value	Q	p value	I^2^	
Breed								
Arbor Acres	89	0.99	[0.71, 1.28]	<0.001	370.90	<0.001	76	<0.001
Cobb	18	0.91	[0.34, 1.48]	0.002	89.25	<0.001	81	
Cuban EB24	6	2.13	[0.85, 3.42]	0.001	39.21	<0.001	87	
Lingnan Yellow	18	0.57	[0.22, 0.91]	0.001	26.11	0.073	35	
Ross	46	1.21	[0.87, 1.54]	<0.001	235.87	<0.001	81	
Sasso X44	8	0.63	[−0.11, 1.38]	0.096	20.77	0.004	66	
Tsukuba jidori	6	−0.17	[−0.68, 0.34]	0.513	2.68	0.749	00	
Unknown	14	1.02	[−0.20, 2.24]	0.102	70.48	<0.001	82	
Sex2)								
Both	30	1.26	[0.88, 1.65]	<0.001	76.17	<0.001	62	0.077
Male	101	1.07	[0.80, 1.33]	<0.001	481.64	<0.001	79	
Unknown	71	0.74	[0.44, 1.03]	<0.001	317.40	<0.001	78	
DFM product								
Commercial	68	0.89	[0.58, 1.19]	<0.001	333.85	<0.001	80	0.570
Non-commercial	137	0.99	[0.78, 1.21]	<0.001	568.70	<0.001	76	
Microbial species								
Multiple	58	0.56	[0.31, 0.81]	<0.001	183.41	<0.001	69	0.004
Single	142	1.09	[0.87, 1.32]	<0.001	636.58	<0.001	78	
Unknown	5	2.38	[0.02, 4.73]	0.048	55.90	<0.001	93	
Sampling organ3)								
Crop	12	1.75	[0.88, 2.63]	<0.001	37.68	<0.001	71	0.386
Ileum	38	0.72	[0.29, 1.15]	0.001	191.67	<0.001	81	
Caecum	110	0.90	[0.68, 1.12]	<0.001	466.25	<0.001	77	
Colon	14	1.23	[0.02, 2.43]	0.046	71.51	<0.001	82	
Excreta	7	1.20	[0.11, 2.28]	0.030	51.95	<0.001	88	
Unknown	21	1.09	[0.52, 1.62]	<0.001	51.45	<0.001	61	
Application route4)								
Feed	191	0.96	[0.77, 1.14]	<0.001	863.92	<0.001	78	0.785
Water	9	0.73	[−0.10, 1.55]	0.085	19.06	0.015	58	
Gavage	4	1.36	[−0.44, 3.16]	0.139	17.08	0.001	82	
Application frequency								
Daily	201	0.95	[0.77, 1.013]	<0.001	885.43	<0.001	77	0.658
Once	4	1.36	[−0.44, 3.16]	0.139	17.08	0.001	82	
Application duration(days of age)								
≤7	29	0.64	[0.31, 0.97]	<0.001	51.01	0.005	45	<0.001
>7, ≤14	26	0.47	[0.11, 0.83]	0.011	58.73	<0.001	57	
>14, ≤21	49	1.05	[0.57, 1.53]	<0.001	285.76	<0.001	83	
>21, ≤42	95	1.23	[0.98, 1.49]	<0.001	477.93	<0.001	80	
>42	6	−0.17	[−0.68, 0.36]	0.513	2.68	0.749	00	

DFM, direct-fed microbial; SMD, standardized mean difference; CI, confidence interval.

1) n, number of comparisons.

2) Three comparisons of “Female” sex were excluded from the subgroup analysis.

3) Three comparisons of “Duodenum” sampling organ were excluded from the subgroup analysis.

4) One comparison of “Feed and water” application route was excluded from the subgroup analysis.

**Table 3:** Subgroup analysis of the effect of DFM supplementation on the log concentrations of coliforms and *Escherichia coli* in broiler chickens

Characteristics and subgroups	n1)	Effect size			Heterogeneity			p value for subgroup difference
		SMD	95% CI	p value	Q	p value	I^2^	
Breed								
Arbor Acres	70	−0.68	[−0.93, −0.43]	<0.001	206.14	<0.001	67	<0.001
Cobb	18	−0.30	[−0.76,0.17]	0.211	65.39	<0.001	74	
Cuban EB24	6	−0.56	[−1.02, −0.11]	0.016	7.55	0.183	34	
Lingnan Yellow	18	−0.82	[−1.31, −0.33]	0.001	47.52	<0.001	64	
Ross	57	−1.61	[−1.97, −1.25]	<0.001	389.77	<0.001	86	
Sasso X44	8	0.06	[−0.36,0.48]	0.792	7.39	0.389	5	
Unknown	12	−5.60	[−7.87, −3.33]	<0.001	66.47	<0.001	83	
Sex								
Both	12	−1.02	[−1.67, −0.37]	0.002	53.42	<0.001	79	0.429
Male	89	−1.10	[−1.36, −0.84]	<0.001	367.03	<0.001	76	
Unknown	88	−0.85	[−1.12, −0.58]	<0.001	422.38	<0.001	79	
DFM product								
Commercial	53	−0.83	[−1.14, −0.53]	<0.001	224.10	<0.001	77	0.273
Non-commercial	136	−1.05	[−1.27, −0.82]	<0.001	640.38	<0.001	79	
Microbial species2)								
Multiple	62	−0.89	[−1.19, −0.60]	<0.001	279.12	<0.001	78	0.639
Single	124	−0.98	[−1.21, −0.75]	<0.001	557.13	<0.001	78	
Sampling organ3)								
Ileum	29	−0.59	[−0.99, −0.19]	0.004	112.50	<0.001	75	<0.001
Cecum	115	−1.04	[−1.27, −0.82]	<0.001	569.28	<0.001	80	
Colon	14	−3.76	[−5.26, −2.27]	<0.001	78.31	<0.001	83	
Excreta	4	−1.23	[−1.95, −0.50]	0.001	7.33	0.062	59	
Unknown	21	−0.39	[−0.78,0.00]	0.052	33.95	0.026	41	
Application route4)								
Feed	178	−1.00	[−1.18, −0.81]	<0.001	832.34	<0.001	79	0.782
Gavage	4	−0.74	[−2.37,0.89]	0.375	15.81	0.001	81	
Water	6	−0.67	[−1.65,0.30]	0.177	13.16	0.022	62	
Application frequency								
Daily	185	−0.98	[−1.16, −0.80]	<0.001	848.39	<0.001	78	0.771
Once	4	−0.74	[−2.37,0.89]	0.375	15.81	0.001	81	
Application duration(days of age)								
≤7	21	−0.16	[−0.48,0.15]	0.318	29.74	0.074	33	<0.001
>7, ≤14	23	−1.07	[−1.62, −0.53]	<0.001	94.20	<0.001	77	
>14, ≤21	47	−0.98	[−1.36, −0.61]	<0.001	196.42	<0.001	77	
>21, ≤42	98	−1.14	[−1.39, −0.88]	<0.001	518.82	<0.001	81	

DFM, direct-fed microbial; SMD, standardized mean difference; CI, confidence interval.

1) n, number of comparisons.

2) Three comparisons of “Unknown” microbial species were excluded from the subgroup analysis.

3) Three comparisons of “Crop” and three comparisons of “Duodenum” sampling organs were excluded from the subgroup analysis.

4) One comparison of “Feed and water” application route was excluded from the subgroup analysis.

**Table 4:** Sensitivity analysis for comparing random-and fixed-effects models

Model	n1)	Effect size			Heterogeneity		
	
		SMD	[95% CI]	p value	Q	p value	I^2^
Random effect							
Category 12)	130	−0.12	[−0.35,0.11]	0.298	592.62	<0.001	78
Category 23)	279	1.07	[0.92,1.22]	<0.001	1,283.64	<0.001	78
Category 34)	290	−1.26	[−1.42, −1.10]	<0.001	1,526.73	<0.001	81
Fixed effect							
Category 1	130	−0.08	[−0.19, 0.02]	0.116	592.62	<0.001	78
Category 2	279	0.77	[0.70, 0.83]	<0.001	1,283.64	<0.001	78
Category 3	290	−0.89	[−0.95, −0.82]	<0.001	1,526.73	<0.001	81

SMD, standardized mean difference; CI, confidence interval.

1) n, number of comparisons.

2) Category 1, bacteria whose log concentrations did not differ significantly from the controls.

3) Category 2, bacteria that their log concentrations increased significantly with DFM supplementation.

4) Category 3, bacteria whose log concentrations decreased significantly with DFM supplementation.
