# Supplementary material for: Effect of direct-fed microbials on culturable gut microbiotas in broiler chickens: a meta-analysis of controlled trials
Source: Asian-Australas J Anim Sci. 2018 May 31;31(11):1781–94. doi: 10.5713/ajas.18.0009 (PMC6212764; doi:10.5713/ajas.18.0009)
Supplement: Supplementary file 1 [file ajas-31-11-1781-supplementary.pdf]

Table S1. Characteristics of the 42 included studies

No.	Author	Breed	Sex	Commercial product (s)	Product name	Microbial species	Species	Strain	Age of treatment	Duration of treatment	Sampling age	Sampling organ	Applied route	Applied frequency	Applied period (days)	Dose
1	Abudabos et al., 2015	Ross 308	M & F	Yes	GalliPro	S	*Bacillus*	*B. subtilis*	1	42	42	Ile, Cae	feed	daily	42	0.2 g GalliPro/kg, GalliPro (8 × 10^{5} CFU *B. subtilis*/g feed)
2	Ahmed et al., 2014	Ross 308	M	No		S	*Bacillus (BAP)*	*Bacillus amyloliquefaciens KB3*	1	35	35	Cae	feed	daily	35	1 g/kg of BAP, 5 g/kg of BAP, 10 g/kg of BAP, 20 g/kg of BAP
3	Altaher et al., 2015	Cobb 500	M	No		M	*Lactobacillus*	*L. Pentosus Ita23 and L. Acidipiscis Ita44*	1	35	35	Cae	feed	daily	35	0.1% of a mixture of *L. pentosus ITA23* and *L. acidophilus ITA44*; 10^{9} cells/kg feed.
4	Boostani et al., 2013-1	Ross 308	M	No		M	*Lactobacillus*	*L. delbrueckii and L. thermophilus*	1	42	21	Cae	water	daily	42	10%, 5% and 2.5 % of water at starter, grower and finisher (yogurt, 1 × 10^{8} cfu of *L. delbrueckii* and *L. thermophilus*)
	Boostani et al., 2013-2						*Sacchromyces and Lactobacillus*	*S. cervisiae and L. acidophilus*								1000, 500 and 250 g/ton feed at starter, grower and finisher (the pax probiotic, 1 × 10^{10} cells/mg of *S. cervisiae* and *L. acidophilus*)
5	Dabiri et al., 2009	Ross 308	"?"	Yes	Primalac	"?"	*"?"*	*"?"*	1	21	21	Cro, Ile	feed	daily	21	900g/ton
6	Daşkiran et al., 2012	Ross 308	M	Yes	Protexin	M	*Lactobacillus, Bifidobacterium, Streptococcus, Enterococcus, Aspergillus and Candida*	*L. plantarum , L. delbrueckii ssp. bulgaricus, L. acidophillus, L. rhamnosus, B. bifidus, S. salivarius ssp. thermophilus, E. faecium, A. oryzae and C. pintolepesii*	1	42	10, 21 ,42	Ile	feed	daily	42	0.5 kg/ton feed, *L. plantarum* (1.89 x 10^{10} cfu/kg), *L. delbrueckii ssp. bulgaricus* (3.09 x 10^{10} cfu/kg), *L. acidophillus* (3.09 x 10^{10} cfu/kg), *L. rhamnosus* (3.09 x 10^{10} cfu/kg), *B. bifidus* (3.00 x 10^{10} cfu/kg), *S. salivarius ssp. thermophilus* (6.15 x 10^{10} cfu/kg), *E. faecium* (8.85 x 10^{10} cfu/kg), *A. oryzae* (7.98 x 10^{10} cfu/kg) and *C. pintolepesii* (7.98 x10^{10} cfu/kg)

Abbreviation: M = Male, F = Female (for “Sex” column); S = single species, M = Multiple species (for “Microbial species” column); Cae = Caecum, Col = Colon, Cro = Crop, Duo = Duodenum, Ile = Ileum, Exc = Excreta (for “Sampling organ” column); “?”= Unknown

Table S 1. (Cont.)

No.	Author	Breed	Sex	Commercial product (s)	Product name	Microbial species	Species	Strain	Age of treatment	Duration of treatment	Sampling age	Sampling organ	Applied route	Applied frequency	Applied period (days)	Dose
7	Deniz et al., 2011	Ross 308	M	No		S	*Bacillus*	*B.subtilis DSM17299*	1	42	42	Cae	feed	daily	42	1g/kg of food (*B.subtilis DSM17299*, 8 x10^5 cfu/kg of food)
8	Fajardo et al., 2012-EX1-1	Sasso X44	"?"	No		S	*Lactobacillus*	*L. casei CECT 4043*	1	42	7, 14, 21, 42	"?"	feed	daily	42	20 ml/kg feed of *L. casei CECT 4043* (3.69 × 10^9 CFU/mL)
	Fajardo et al., 2012-EX1-2							*L. lactis CECT 539*								20 ml/kg feed of *L. lactis CECT 539* (3.34 × 10^9 CFU/mL)
	Fajardo et al., 2012-Ex-2-1	Ross 308						*L. casei CECT 4043*		31	7, 16, 31	Cae			31	20 ml/kg feed of *L. casei CECT 4043* (3.69 × 10^9 CFU/mL)
	Fajardo et al., 2012-Ex-2-2							*L. lactis CECT 539*								20 ml/kg feed of *L. lactis CECT 539* (3.34 × 10^9 CFU/mL)
9	Fujiwara et al., 2009-1	Tsukuba jidori	M	Yes	"?"	S	*Bacillus*	*B. subtilis var.natto*	0	80	80	Cae	feed	daily	80	1% of Fermented Soybean (*B. subtilis* var.natto, 1 x 10^9 cfu/g)
	Fujiwara et al., 2009-2															2% of Fermented Soybean (*B. subtilis* var.natto, 1 x 10^9 cfu/g)
	Fujiwara et al., 2009-3								28	53	80	Cae			53	2% of Fermented Soybean (*B. subtilis* var.natto, 1 x 10^9 cfu/g)
	Fujiwara et al., 2009-1		F						0	80	80	Cae			80	1% of Fermented Soybean (*B. subtilis* var.natto, 1 x 10^9 cfu/g)
	Fujiwara et al., 2009-2															2% of Fermented Soybean (*B. subtilis* var.natto, 1 x 10^9 cfu/g)
	Fujiwara et al., 2009-3								28	53	80				53	2% of Fermented Soybean (*B. subtilis* var.natto, 1 x 10^9 cfu/g)

Table S 1. (Cont.)

No.	Author	Breed	Sex	Commercial product (s)	Product name	Microbial species	Species	Strain	Age of treatment	Duration of treatment	Sampling age	Sampling organ	Applied route	Applied frequency	Applied period (days)	Dose
10	Gerard et al., 2008-1	Ross	"?"	No		S	*Lactobacillus*	*Lactobacillus sp. No. I-2673*	1	19	4, 19	Cae	water	daily	19	Drinking water of *Lactobacillus* sp. No. I-2673 (2.7 x 10^6 CFU/ml)
11	Jeong and Kim, 2014-1	Ross 308	M	Yes	Calsporin	S	*Bacillus*	*B. subtilis C-3102*	1	35	35	Ile, Cae, Col, Exc	feed	daily	35	Basal diet + 300 mg of *B. subtilis*/kg of feed (1×10^9 cfu/kg)
	Jeong and Kim, 2014-2															Basal diet + 600 mg of *B. subtilis*/kg of feed (1×10^9 cfu/kg)
12	Jin et al., 1998a-1	Arbor Acre	"?"	No		S	*Lactobacillus*	*L. acidophilus I 26*	1	42	10, 20, 30, 40	Ile, Cae	feed	daily	42	basal diet + 1 g/ kg *L. acidophilus* I 26, (viable cells 1 x 10^9 cells/g)
	Jin et al., 1998a-2					M		*12 strains of Lactobacillus (2 strains of L. acidophilus, 3 strains of L. fermentum, 1 strain of L. crispatus and 6 strains of L. breĺis)*								basal diet + 1g/ kg mixture *Lactobacillus*, (viable cells 1 x 10^9 cells/g)
13	Jin et al., 1996-1	Arbor Acres	M	No		S	*Bacillus*	*B. sutilis*	10	38	17, 24, 31, 38	"?"	feed	daily	38	Basal diet + 0.1% of *B. sutilis* (1 x 10^9 living microorganism/g)
	Jin et al., 1996-2						*Lactobacilli culture*	*Lactobacilli*								Basal diet + 0.2% of *Lactobacillus* (0.5-1 x 10^9 viable cell/g)
	Jin et al., 1996-3															5g of *Lactobacillus* in Drinking water/cage/day (0.5-1 x 10^9 viable cell/g)
14	Jin et al., 1998b-1	Arbor Acre	"?"	No		M	*Four species (L. acidophilus, L. fermentum, L. crispatus, and L. brevis)*	*Twelve strains of Lactobacillus*	1	42	10, 20, 30, 40	Ile, Cae	feed	daily	42	0.05% of *Lactobacillus* culture (LC), 1 x 10^9 cell/g
	Jin et al., 1998b-2															0.10% of *Lactobacillus* culture (LC), 1 x 10^9 cell/g
	Jin et al., 1998b-3															0.15% of Lactobacillus culture (LC), 1 x 10^9 cell/g

Table S 1. (Cont.)

No.	Author	Breed	Sex	Commercial product (s)	Product name	Microbial species	Species	Strain	Age of treatment	Duration of treatment	Sampling age	Sampling organ	Applied route	Applied frequency	Applied period (days)	Dose
15	Jung et al., 2008	"?"	M	Yes	Abiasa (company's name)	S	*Bifidobacterium*	*B. lactis D300*	1	40	7, 40	Exc	feed	daily	40	0.15 kg product /25 kg of diet, (300 × 109 cells/g)
16	Kim et al., 2012-1	Ross	"?"	No		M	*Lactobacillus, Bacillus, Saccharomyces*	*L. acidophilus, B. subtilis and S. cerevisiae*	1	35	21, 35	Cae	feed	daily	35	10^7 cfu multi-microbe probiotic/kg of diet
	Kim et al., 2012-2															10^8 cfu multi-microbe probiotic/kg of diet
	Kim et al., 2012-3															10^9 cfu multi-microbe probiotic/kg of diet
17	Lei et al., 2015-1	Arbor Acres	M	No		S	*Bacillus*	*Bacillus amyloliquefaciens*	1	42	21, 42	Cae	feed	daily	42	30 mg/kg (7.5×10^7 cfu/kg) of DFM
	Lei et al., 2015-2															60 mg/kg (1.5×10^8 cfu/kg) of DFM
18	Lin et al., 2011-1	Arbor Acres	"?"	No		S	*Bacillus*	*B. coagulans*	1	42	42	Duo, Cae	feed	daily	42	Basal diet + 0.005% (*B. caogulans*, 8 $\log_{10}$ cfu/g)
	Lin et al., 2011-2															Basal diet + 0.02% (*B. caogulans*, 8 $\log_{10}$ cfu/g)
	Lin et al., 2011-3															Basal diet + 0.04% (*B. caogulans*, 8 $\log_{10}$ cfu/g)
19	Liu et al., 2007	Arbor Acres	M&F	No		S	*Lactobacillus*	*L. reuteri Pg4*	1	37	21, 37	Ile, Cae	feed	daily	37	1 g *L. reuteri Pg4* powder/kg feed, (10^9 cfu/g)
20	Milián et al., 2013-1	Cuban EB24	"?"	No		S	*Bacillus*	*B. subtilis*	1	42	21, 42	Cae	feed	daily	42	Dosage of 10^9 endospores/g of concentrates (biopreparation C-31)
	Milián et al., 2013-2															Dosage of 10^9 endospores/g of concentrates (biopreparation C-34)
	Milián et al., 2013-3															Dosage of 10^9 endospores/g of concentrates (biopreparation E-44)

Table S 1. (Cont.)

No.	Author	Breed	Sex	Commercial product (s)	Product name	Microbial species	Species	Strain	Age of treatment	Duration of treatment	Sampling age	Sampling organ	Applied route	Applied frequency	Applied period (days)	Dose
21	Mohammadi Gheisar et al., 2016-1	Ross 308	M&F	Yes	Lactiferm	S	*Enterococcus*	*E. faecium M74*	1	35	35	Exc	feed	daily	35	0.25% probiotic (3 × 10^{11} CFU/g) of feed
	Mohammadi Gheisar et al., 2016-2															0.50% probiotic (3 × 10^{11} CFU/g) of feed
22	Mookiah et al., 2014	Ross 308	M	No		M	*Lactobacillus*	*The 11 Lactobacillus strains (L. reuteri C 1, C 10 and C 16; L. gallinarum I 16 and I 26; L. brevis I 12, I 23, I 25, I 218 and I 211, and L. salivarius I 24)*	1	42	21, 42	Cae	feed	daily	42	1g of probiotic/kg feed
23	Mountzouris et al., 2015	Cobb	M	Yes	Levucell SB 20	S	*Saccharomyces cerevisae var. boulardii CNCM I-1079*	*S. cerevisae var. boulardii CNCM I-1079*	1	35	15, 35	Cae	feed	daily	35	50 mg /kg diet (*S. cerevisae*, 1 × 10^{9} cfu)
24	Mountzouris et al., 2007-1	Cobb	M	Yes	Biomin Poultry5Star	M	*Lactobacillus, Enterococcus, Bifidobacterium, Pediococcus*	*L. reuteri, L. salivarius, E. faecium, B. animalis and P. acidilactici*	1	42	42	Cae	feed and water	daily	42	1 g/kg of feed and 1 g/kg in water (feed= 6weeks, Water= first 4weeks, 2 × 10^{12} cfu/kg of product)
	Mountzouris et al., 2007-2												feed			1 g/kg of feed, 2 × 10^{12} cfu/kg of product.
25	Mountzouris et al., 2010-1	Cobb	M	Yes	PoultryStar ME	M	*Lactobacillus, Enterococcus, Bifidobacterium, Pediococcus*	*L. reuteri DSM 16350, L. salivarius DSM 16351, E. faecium DSM 16211, B. animalis DSM 16284 and P. acidilactici DSM 16210*	1	42	14, 28, 42	Cae	feed	daily	42	1 g/kg of diet, 1 × 10^{8} cfu/kg of product.
	Mountzouris et al., 2010-2															1 g/kg of diet, 1 × 10^{9} cfu/kg of product.
	Mountzouris et al., 2010-3															1 g/kg of diet, 1 × 10^{10} cfu/kg of product.

Table S 1. (Cont.)

No.	Author	Breed	Sex	Commercial product (s)	Product name	Microbial species	Species	Strain	Age of treatment	Duration of treatment	Sampling age	Sampling organ	Applied route	Applied frequency	Applied period (days)	Dose
26	Park and Kim, 2014-1	Ross 308	M	No		S	*Bacillus*	*Bacillus subtilis B2A*	1	28	28	Col, "?"	feed	daily	28	*B. subtilis B2A* 1.1×10^4 cfu/g of feed.
	Park and Kim, 2014-2															*B. subtilis B2A* 1.0×10^5 cfu/g of feed.
	Park and Kim, 2014-3															*B. subtilis B2A* 1.0×10^6 cfu/g of feed.
27	Peng et al., 2016-1	Arbor Acres	"?"	No		S	*Lactobacillus*	*L. plantarum B1*	1	21	21	Cae	feed	daily	21	*L. plantarum B1* (2×10^9 cfu/kg), from 1-21 day
	Peng et al., 2016-2								22		42					*L. plantarum B1* (2×10^9 cfu/kg), from 22-42 day
	Peng et al., 2016-3								1	42	21, 42				42	*L. plantarum B1* (2×10^9 cfu/kg), from 1-42 day
28	Rada and Marounek, 1997	Ross	"?"	No		S	*Lactobacillus*	*L. salivarus 51R*	1-6h	5	1, 5	Cro, Cae	gavage	once	5	0.2 ml (5×10^8 cfu/ml)
29	Rodriguez et al., 2012	Cobb	M	Yes	Cylactin	S	*Enterococcus*	*E. faecium*	1	28	28	Ile, Cae	feed	daily	28	Cylactin (2 g/kg basal diet)
30	Salim et al., 2013-1	Ross 308	M	Yes	"?"	S	*Lactobacillus*	*L. reuteri*	1	35	35	Cae	feed	daily	35	0.1% DFM in feed (*L. reuteri*)
	Salim et al., 2013-2					M	*Lactobacillus, Bacillus and Saccharomyces*	*L. reuteri, B. subtilis, and S. cerevisiae*								0.1% DFM in feed (*L. reuteri*, *B. subtilis*, and *S. cerevisiae*)
31	Samli et al., 2007	Ross 308	M	Yes	CYLACTIN	S	*Enterococcus*	*E. faecium NCIMB 10415*	1	21	21	Ile, Exc	feed	daily	21	1×10^{10} cfu/g
32	Sen et al., 2012-1	Ross	"?"	No		S	*Bacillus*	*Bacillus subtilis LS 1-2*	1	35	21, 35	Cae	feed	daily	35	0.15% of *B. subtilis* LS 1-2.
	Sen et al., 2012-2															0.30% of *B. subtilis* LS 1-2.
	Sen et al., 2012-3															0.45% of *B. subtilis* LS 1-2.
33	Shams Shargh et al., 2012	Ross 308	M	Yes	Protexin	M	*Lactobacillus, Streptococcus, Aspergillus, Bifidobacterium, Enterococcus, and Candida*	*L. acidophilus, L. plantarum, L. rhamnosus, L. bulgaricus, S. thermophilus, A. oryzae, B. bifidum, E. faecium, and C. pintolepesii*	1	42	20, 40	Cro, Cae	water	daily	42	Protexin (1,000 ppm for 0-7d, 500 ppm for 8-21d and 250 ppm for 22-42d)

Table S 1. (Cont.)

No.	Author	Breed	Sex	Commercial product (s)	Product name	Microbial species	Species	Strain	Age of treatment	Duration of treatment	Sampling age	Sampling organ	Applied route	Applied frequency	Applied period (days)	Dose
34	Shen et al., 2014	Cobb500	M&F	No		S	*Lactobacillus*	*L. plantarum*	1	42	21, 42	Cae	feed	daily	42	Basal diet 1 g/kg (0.1%, m/m), 1 × 10^9 cfu/mL
35	Shim et al., 2012-1	Ross 308	M	No		M	*Lactobacillus, Bacillus, Saccharomyces and Aspergillus*	*L. acidophilus, B. sutilis, S. cerevisiae, A. oryzae*	1	35	21, 35	Cae	feed	daily	35	Basal diet + 0.30% of LT probiotic (*L. acidophilus* (4.0 x 10^8 cfu/g), *B. sutilis* (4.8 x 10^9 cfu/g), *S. cerevisiae* (1.0 x 10^4 cfu/g) and *A. oryzae* (4.3 x 10^7 cfu/g)
	Shim et al., 2012-2															Basal diet + 0.30% of LT probiotic (*L. acidophilus* (1.0 x 10^2 cfu/g), *B. sutilis* (2.0 x 10^4 cfu/g), *S. cerevisiae* (1.2 x 10^2 cfu/g) and *A. oryzae* (1.0 x 10^3 cfu/g)
36	Wu et al., 2011-1	"?"	M	No		S	*Bacillus*	*Bacillus sp. KD1 and Bacillus sp. YB*	1	42	21, 42	Col	feed	daily	42	*Bacillus sp. KD1* 0.05% of diet weight, (10^9 bacilli/kg of feed)
	Wu et al., 2011-2															*Bacillus sp. KD1* 0.25% of diet weight, (5 × 10^9 bacilli/kg of feed)
	Wu et al., 2011-3															*Bacillus sp. KD1* 0.5% of diet weight, (10^{10} bacilli/kg of feed)
	Wu et al., 2011-4															*Bacillus sp. YB* 0.05% of diet weight, (10^9 bacilli/kg of feed)
	Wu et al., 2011-5															*Bacillus sp. YB* 0.25% of diet weight, (5 × 10^9 bacilli/kg of feed)
	Wu et al., 2011-6															*Bacillus sp. YB* 0.5% of diet weight, (10^{10} bacilli/kg of feed)
37	Yakhkeshi et al., 2012	Ross 308	M	Yes	Primalac	"?"			1	42	42	Cro, Ile, Cae	feed	daily	42	0.1% of diet

Table S 1. (Cont.)

No.	Author	Breed	Sex	Commercial product (s)	Product name	Microbial species	Species	Strain	Age of treatment	Duration of treatment	Sampling age	Sampling organ	Applied route	Applied frequency	Applied period (days)	Dose
38	Yanget al., 2012-1	Lingnan Yellow	M	Yes	"?"	S	*Clostridium*	*C. butyricum HJCB998*	1	42	3, 5, 7, 14, 21, 42	Cae	feed	daily	42	Basal diet containing 1 × 10^7 cfu *C. Butyricum*/kg
	Yanget al., 2012-2							*C. butyricum HJCB998*								Basal diet containing 2 × 10^7 cfu *C. Butyricum*/kg
	Yanget al., 2012-3							*C. butyricum HJCB998*								Basal diet containing 3 × 10^7 cfu *C. Butyricum*/kg
39	Yu et al., 2007	Arbor Acres	M&F	No		S	*Lactobacillus*	*L. reuteri Pg4*	1	42	1, 3, 7, 14, 28, 42	Cro, Ile, Cae	feed	daily	42	0.1% L. reuteri Pg4 powder (10^8 CFU/g)
40	Zhang et al., 2016-1	Ross 308	M	No		S	*Lactococcus*	*L. garvieae B301*	1	28	14	Cae	feed	daily	28	Basal diet plus 3.36 x 10^9 cfu/kg of L. garvieae B301
	Zhang et al., 2016-2										28					Basal diet plus 3.36 x 10^9 cfu/kg of L. garvieae B301
41	Zhang et al., 2013	Arbor Acres	M&F	No		S	*Bacillus*	*B. subtilis UBT-MO_2*	1	35	35	Cae, "?"	feed	daily	35	10^5 cfu/kg of feed
42	Zhang and Kim, 2014-1	Ross 308	M&F	Yes	Probion	M	*Lactobacillus, Bacillus, Clostridium*	*L. acidophilus, B. subtilis DSM 17299, and C. butyricum*	1	35	35	Cae	feed	daily	35	Starter diet 1.07 × 10^5 cfu/kg and Grower diet 1.12 × 10^5 cfu/kg
	Zhang and Kim, 2014-2															Starter diet 2.11 × 10^5 cfu/kg and Grower diet 2.09 × 10^5 cfu/kg

Table S2. Risk of bias assessment

Authors\RoB	1.Was the allocation sequence adequately generated and applied?	2.Were the groups similar at baseline or were they adjusted for confounders in the analysis?	3.Was the allocation adequately concealed?	4.Were the animals randomly housed during the experiment?	5.Were the caregivers and/or investigators blinded from knowledge which intervention each animal received during the experiment?	6. Were animals selected at random for outcome assessment?	7.Was the outcome assessor blinded?	8.Were incomplete outcome data adequately addressed?	9.Are reports of the study free of selective outcome reporting?	10.Was the study apparently free of other problems that could result in high risk of bias?
Abudabos et al., 2015	No	Yes	?	Yes	?	No	Yes	Yes	?	?
Ahmed et al., 2014	?	Yes	?	Yes	?	Yes	Yes	Yes	?	?
Altaher et al., 2015	?	Yes	?	?	?	No	Yes	Yes	?	?
Boostani et al., 2013	?	Yes	?	Yes	?	Yes	Yes	Yes	?	?
Dabiri et al., 2009	?	?	?	?	?	No	Yes	Yes	?	?
Daşkiran et al., 2012	?	Yes	?	?	?	Yes	Yes	Yes	?	?
Deniz et al., 2011	?	Yes	?	?	?	No	Yes	Yes	?	?
Fajardo et al., 2012	No	?	?	?	?	Yes	Yes	Yes	?	?
Fujiwara et al., 2009	?	Yes	?	?	?	Yes	Yes	Yes	?	?
Gerard et al., 2008	?	?	?	?	?	No	Yes	?	?	?
Jeong and Kim, 2014	?	Yes	?	Yes	?	Yes	Yes	Yes	?	?
Jin et al., 1998a	?	?	?	?	?	Yes	Yes	Yes	?	?
Jin et al., 1996	?	?	?	?	?	Yes	Yes	Yes	?	?
Jin et al., 1998b	?	?	?	?	?	Yes	Yes	Yes	?	?
Jung et al., 2008	?	?	?	?	?	Yes	Yes	Yes	?	?
Kim et al., 2012	?	?	?	?	?	No	Yes	Yes	?	?
Lei et al., 2015	?	Yes	?	Yes	?	Yes	Yes	Yes	?	?
Lin et al., 2011	?	?	?	?	?	No	Yes	Yes	?	?
Liu et al., 2007	?	?	?	?	?	No	Yes	Yes	?	?
Milián et al., 2013	?	?	?	?	?	No	Yes	Yes	?	?

Mohammadi Gheisar et al., 2016	No	?	No	?	?	Yes	Yes	Yes	?	?
Authors\RoB	1.Was the allocation sequence adequately generated and applied?	2.Were the groups similar at baseline or were they adjusted for confounders in the analysis?	3.Was the allocation adequately concealed?	4.Were the animals randomly housed during the experiment?	5.Were the caregivers and/or investigators blinded from knowledge which intervention each animal received during the experiment?	6. Were animals selected at random for outcome assessment?	7.Was the outcome assessor blinded?	8.Were incomplete outcome data adequately addressed?	9.Are reports of the study free of selective outcome reporting?	10.Was the study apparently free of other problems that could result in high risk of bias?
Mookiah et al., 2014	?	Yes	?	?	?	Yes	Yes	Yes	?	?
Mountzouris et al., 2015	?	Yes	?	Yes	?	No	Yes	Yes	?	?
Mountzouris et al., 2007	?	Yes	?	?	?	Yes	Yes	Yes	?	?
Mountzouris et al., 2010	?	Yes	?	?	?	Yes	Yes	Yes	?	?
Park and Kim, 2014	No	Yes	?	?	?	No	Yes	Yes	?	?
Peng et al., 2016	?	?	?	?	?	Yes	Yes	Yes	?	?
Rada and Marounek, 1997	No	Yes	?	?	?	No	Yes	Yes	?	?
Rodriguez et al., 2012	No	Yes	?	?	?	No	Yes	Yes	?	?
Salim et al., 2013	?	Yes	?	Yes	?	No	Yes	Yes	?	?
Samli et al., 2007	?	Yes	?	?	?	Yes	Yes	Yes	?	?
Sen et al., 2012	?	?	?	?	?	No	Yes	Yes	?	?
Shams Shargh et al., 2012	?	Yes	?	Yes	?	No	Yes	Yes	?	?
Shen et al., 2014	?	?	?	?	?	Yes	Yes	Yes	?	?
Shim et al., 2012	?	Yes	?	?	?	No	Yes	Yes	?	?
Wu et al., 2011	?	?	?	Yes	?	Yes	Yes	Yes	?	?
Yakhkeshi et al., 2012	?	Yes	?	?	?	Yes	Yes	Yes	?	?
Yang et al., 2012	?	Yes	?	Yes	?	No	Yes	Yes	?	?
Yu et al., 2007	?	?	?	Yes	?	No	Yes	Yes	?	?
Zhang et al., 2016	?	Yes	?	?	?	Yes	Yes	Yes	?	?
Zhang et al., 2013	?	?	?	?	?	Yes	Yes	Yes	?	?
Zhang and Kim, 2014	?	?	?	Yes	?	No	Yes	Yes	?	?

Table S3. Additional subgroup analysis of DFM genus on the log concentrations of *Lactobacillus* in broiler chickens

Characteristics and subgroups	Effect size				Heterogeneity			p value for subgroup difference
	n[1)]	SMD	[95% CI]	p value	Q	p value	I^2	
DFM genus[2)]								
Bacillus	55	1.17	[0.80, 1.54]	<0.001	294.29	<0.001	82	0.016
Clostridium	18	0.57	[0.22, 0.91]	0.001	26.11	0.073	35	
Enterococcus	6	2.20	[0.55, 3.85]	0.009	90.25	<0.001	94	
Lactobacillus	59	1.22	[0.87, 1.57]	<0.001	191.50	<0.001	70	
Overall	138	0.99	[0.79, 1.20]	<0.001	636.58	<0.001	78	

CI, confidence interval; SMD, standardized mean difference.

[1)] n, number of comparisons.

[2)] genus with less than four comparisons, multiple species, unknown species of DFM were excluded from the subgroup analysis.

Table S4. Additional subgroup analysis of DFM genus on the log concentrations of coliforms and *Escherichia coli* in broiler chickens

Characteristics and subgroups	Effect size				Heterogeneity			p value for subgroup difference
	n[1)]	SMD	[95% CI]	p value	Q	p value	I^2	
DFM genus[2)]								
Bacillus	53	-1.55	[-1.93, -1.18]	<0.001	269.27	<0.001	81	0.001
Clostridium	18	-0.82	[-1.31, -0.33]	0.001	47.52	<0.001	64	
Enterococcus	4	-0.45	[-1.20, 0.29]	0.234	11.87	0.008	75	
Lactobacillus	47	-0.59	[-0.96, -0.23]	0.002	178.66	<0.001	74	
Overall	122	-0.96	[-1.18, -0.74]	<0.001	543.21	<0.001	78	

CI, confidence interval; SMD, standardized mean difference.

[1)] n, number of comparisons.

[2)] genus with less than four comparisons, multiple species, unknown species of DFM were excluded from the subgroup analysis.

Table S5. References for all included studies.

No.	References
1.	Abudabos AM, Al-Batshan HA, Murshed MA. Effects of prebiotics and probiotics on the performance and bacterial colonization of broiler chickens. S Afr J Anim Sci 2015;45:419-28.
2.	Ahmed ST, Islam M, Mun HS, Sim HJ, Kim YJ, Yang CJ. Effects of *Bacillus amyloliquefaciens* as a probiotic strain on growth performance, cecal microflora, and fecal noxious gas emissions of broiler chickens. Poult Sci 2014;93:1963-71.
3.	Altaher YW, Jahromi MF, Ebrahim R, Zulkifli I, Liang JB. *Lactobacillus pentosus* Ita23 and *L. Acidipiscis* Ita44 enhance feed conversion efficiency and beneficial gut microbiota in broiler chickens. Rev Bras Cienc Avic 2015;17:159-64.
4.	Boostani A, Mahmoodian Fard HR, Ashayerizadeh A, Aminafshar M. Growth performance, carcass yield and intestinal microflora populations of broilers fed diets containing thepax and yogurt. Rev Bras Cienc Avic 2013;15:1-6.
5.	Dabiri N, Ashayerizadeh A, Ashayerizadeh O, et al. Comparison effects of several growth stimulating additives on performance responses and microbial population in crop and ileum of broiler chickens on their 21st day of life. J Anim Vet Adv 2009;8:1509-15.
6.	Daşkiran M, Öno AG, Cengiz Ö, et al. Influence of dietary probiotic inclusion on growth performance, blood parameters, and intestinal microflora of male broiler chickens exposed to posthatch holding time. J Appl Poult Res 2012;21:612-22.
7.	Deniz G, Orman A, Cetinkaya F, Gencoglu H, Meral Y, Turkmen II. Effects of probiotic (*Bacillus subtilis* DSM 17299) supplementation on the caecal microflora and performance in broiler chickens. Rev Med Vet (Toulouse) 2011;162:538-45.
8.	Fajardo P, Pastrana L, Méndez J, Rodríguez I, Fucios C, Guerra NP. Effects of feeding of two potentially probiotic preparations from lactic acid bacteria on the performance and faecal microflora of broiler chickens. Sci World J 2012;2012:562635.
9.	Fujiwara K, Yamazaki M, Abe H, et al. Effect of *Bacillus subtilis* var. natto fermented soybean on growth performance, microbial activity in the caeca and cytokine gene expression of domestic meat type chickens. J Poult Sci 2009;46:116-22.
10.	Gerard P, Brezillon C, Quere F, Salmon A, Rabot S. Characterization of cecal microbiota and response to an orally administered *Lactobacillus* probiotic strain in the broiler chicken. J Mol Microbiol Biotechnol 2008;14:115-22.
11.	Jeong JS, Kim IH. Effect of *Bacillus subtilis* C-3102 spores as a probiotic feed supplement on growth performance, noxious gas emission, and intestinal microflora in broilers. Poult Sci 2014;93:3097-103.
12.	Jin LZ, Ho YW, Abdullah N, Ali MA, Jalaludin S. Effects of adherent *Lactobacillus* cultures on growth, weight of organs and intestinal microflora and volatile fatty acids in broilers. Anim Feed Sci Technol 1998;70:197-209.
13.	Jin LZ, Ho YW, Abdullah N, Jalaludin S. Influence of dried *Bacillus subtilis* and lactobacilli cultures on intestinal microflora and performance in broilers. Asian-Australas J Anim Sci 1996;9:397-403.
14.	Jin LZ, Ho YW, Abdullah N, Jalaludin S. Growth performance, intestinal microbial populations, and serum cholesterol of broilers fed diets containing Lactobacillus cultures. Poult Sci 1998;77:1259-65.
15.	Jung SJ, Houde R, Baurhoo B, Zhao X, Lee BH. Effects of galacto-oligosaccharides and a *Bifidobacteria lactis*-based probiotic strain on the growth performance and fecal microflora of broiler chickens. Poult Sci 2008;87:1694-9.
16.	Kim JS, Ingale SL, Kim YW, et al. Effect of supplementation of multi-microbe probiotic product on growth performance, apparent digestibility, cecal microbiota and small intestinal morphology of broilers. J Anim Physiol Anim Nutr (Berl) 2012;96:618-26.
17.	Lei X, Piao X, Ru Y, Zhang H, Peron A, Zhang H. Effect of *Bacillus amyloliquefaciens*-based direct-fed microbial on performance, nutrient utilization, intestinal morphology and cecal microflora in broiler chickens. Asian-Australas J Anim Sci 2015;28:239-46.
18.	Lin SY, Hung ATY, Lu JJ. Effects of supplement with different level of *Bacillus coagulans* as probiotics on growth performance and intestinal microflora populations of broiler chickens. J Anim Vet Adv 2011;10:111-4.
19.	Liu JR, Lai SF, Yu B. Evaluation of an intestinal *Lactobacillus reuteri* strain expressing rumen fungal xylanase as a probiotic for broiler chickens fed on a wheat-based diet. Br Poult Sci 2007;48:507-14.
20.	Milián G, Rondón AJ, Pérez M, et al. Evaluation of *Bacillus subtilis* biopreparations as growth promoters in chickens. Cuban J Agri Sci 2013;47:61-6.
21.	Mohammadi Gheisar M, Hosseindoust A, Kim IH. Effects of dietary *Enterococcus faecium* on growth performance, carcass characteristics, faecal microbiota, and blood profile in broilers. Vet Med (Praha) 2016;61:28-34.

22.	Mookiah S, Sieo CC, Ramasamy K, Abdullah N, Ho YW. Effects of dietary prebiotics, probiotic and synbiotics on performance, caecal bacterial populations and caecal fermentation concentrations of broiler chickens. J Sci Food Agric 2014;94:341-8.
23.	Mountzouris KC, Dalaka E, Palamidi I, et al. Evaluation of yeast dietary supplementation in broilers challenged or not with *Salmonella* on growth performance, cecal microbiota composition and *Salmonella* in ceca, cloacae and carcass skin. Poult Sci 2015;94:2445-55.
24.	Mountzouris KC, Tsirtsikos P, Kalamara E, Nitsch S, Schatzmayr G, Fegeros K. Evaluation of the efficacy of a probiotic containing *Lactobacillus*, *Bifidobacterium*, *Enterococcus*, and *Pediococcus* strains in promoting broiler performance and modulating cecal microflora composition and metabolic activities. Poult Sci 2007;86:309-17.
25.	Mountzouris KC, Tsitrsikos P, Palamidi I, et al. Effects of probiotic inclusion levels in broiler nutrition on growth performance, nutrient digestibility, plasma immunoglobulins, and cecal microflora composition. Poult Sci 2010;89:58-67.
26.	Park JH, Kim IH. Supplemental effect of probiotic *Bacillus subtilis* B2A on productivity, organ weight, intestinal *Salmonella* microflora, and breast meat quality of growing broiler chicks. Poult Sci 2014;93:2054-9.
27.	Peng Q, Zeng XF, Zhu JL, et al. Effects of dietary *Lactobacillus plantarum* B1 on growth performance, intestinal microbiota, and short chain fatty acid profiles in broiler chickens. Poult Sci 2016;95:893-900.
28.	Rada V, Marounek M. Effect of maduramicin and monensin on survival of *Lactobacillus salivarius* 51R administered in the crop and caeca of young chickens. Arch Tierernahr 1997;50:25-9.
29.	Rodriguez ML, Rebole A, Velasco S, Ortiz LT, Trevino J, Alzueta C. Wheat- and barley-based diets with or without additives influence broiler chicken performance, nutrient digestibility and intestinal microflora. J Sci Food Agric 2012;92:184-90.
30.	Salim HM, Kang HK, Akter N, et al. Supplementation of direct-fed microbials as an alternative to antibiotic on growth performance, immune response, cecal microbial population, and ileal morphology of broiler chickens. Poult Sci 2013;92:2084-90.
31.	Samli HE, Senkoylu N, Koc F, Kanter M, Agma A. Effects of *Enterococcus faecium* and dried whey on broiler performance, gut histomorphology and intestinal microbiota. Arch Anim Nutr 2007;61:42-9.
32.	Sen S, Ingale SL, Kim YW, et al. Effect of supplementation of *Bacillus subtilis* LS 1-2 to broiler diets on growth performance, nutrient retention, caecal microbiology and small intestinal morphology. Res Vet Sci 2012;93:264-8.
33.	Shams Shargh M, Dastar B, Zerehdaran S, Khomeiri M, Moradi A. Effects of using plant extracts and a probiotic on performance, intestinal morphology, and microflora population in broilers. J Appl Poult Res 2012;21:201-8.
34.	Shen X, Yi D, Ni X, et al. Effects of Lactobacillus plantarum on production performance, immune characteristics, antioxidant status, and intestinal microflora of bursin-immunized broilers. Can J Microbiol 2014;60:193-202.
35.	Shim YH, Ingale SL, Kim JS, et al. A multi-microbe probiotic formulation processed at low and high drying temperatures: effects on growth performance, nutrient retention and caecal microbiology of broilers. Br Poult Sci 2012;53:482-90.
36.	Wu BQ, Zhang T, Guo LQ, Lin JF. Effects of *Bacillus subtilis* KD1 on broiler intestinal flora. Poult Sci 2011;90:2493-9.
37.	Yakhkeshi S, Rahimi S, Hemati Matin HR. Effects of yarrow (Achillea millefolium L.), antibiotic and probiotic on performance, immune response, serum lipids and microbial population of broilers. J Agri Sci Tech 2012;14:799-810.
38.	Yang CM, Cao GT, Ferket PR, et al. Effects of probiotic, *Clostridium butyricum*, on growth performance, immune function, and cecal microflora in broiler chickens. Poult Sci 2012;91:2121-9.
39.	Yu B, Liu JR, Chiou MY, Hsu YR, Chiou PWS. The effects of probiotic *Lactobacillus reuteri* Pg4 strain on intestinal characteristics and performance in broilers. Asian-Australas J Anim Sci 2007;20:1243-51.
40.	Zhang T, Xie J, Zhang M, Fu N, Zhang Y. Effect of a potential probiotics *Lactococcus garvieae* B301 on the growth performance, immune parameters and caecum microflora of broiler chickens. J Anim Physiol Anim Nutr (Berl) 2016;100:413-21.
41.	Zhang ZF, Cho JH, Kim IH. Effects of *Bacillus subtilis* UBT-MO2 on growth performance, relative immune organ weight, gas concentration in excreta, and intestinal microbial shedding in broiler chickens. Livest Sci 2013;155:343-7.
42.	Zhang ZF, Kim IH. Effects of multistrain probiotics on growth performance, apparent ileal nutrient digestibility, blood characteristics, cecal microbial shedding, and excreta odor contents in broilers. Poult Sci 2014;93:364-70.

Study name	Statistics for each study				
	Std diff in means	Lower limit	Upper limit	Z-Value	p-Value
Abudabos et al., 2015-1	1.54	0.43	2.66	2.71	0.007
Abudabos et al., 2015-2	-1.41	-2.51	-0.32	-2.53	0.011
Ahmed et al., 2014-1	0.52	-0.02	1.05	1.90	0.057
Ahmed et al., 2014-2	0.11	-0.41	0.64	0.41	0.679
Ahmed et al., 2014-3	0.42	-0.11	0.95	1.55	0.121
Ahmed et al., 2014-4	0.81	0.27	1.36	2.92	0.003
Deniz et al., 2011	2.34	1.55	3.12	5.84	0.000
Zhang et al., 2016-1	0.15	-0.73	1.02	0.33	0.744
Zhang et al., 2016-2	0.20	-0.67	1.08	0.45	0.649
	0.54	0.01	1.06	2.00	0.045

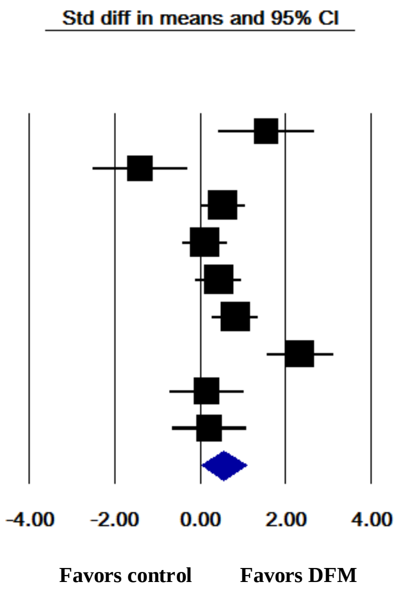

Figure S1. Forest plot for the effect of DFM supplementation on the log concentrations of *Bacillus* in the gut of broiler chickens.

Study name	Std diff in means	Lower limit	Upper limit	Z-Value	p-Value
Jin et al., 1998a-1	1.62	0.20	3.05	2.23	0.026
Jin et al., 1998a-2	1.71	0.26	3.16	2.32	0.021
Jin et al., 1998a-3	-0.90	-2.20	0.40	-1.36	0.174
Jin et al., 1998a-4	0.60	-0.66	1.87	0.93	0.352
Jung et al., 2008-1	1.05	0.12	1.99	2.21	0.027
Jung et al., 2008-2	0.75	-0.16	1.65	1.61	0.107
Kim et al., 2012-1	2.12	0.90	3.35	3.40	0.001
Kim et al., 2012-2	3.53	1.97	5.10	4.42	0.000
Kim et al., 2012-3	1.77	0.61	2.92	3.00	0.003
Kim et al., 2012-4	0.47	-0.52	1.47	0.93	0.352
Kim et al., 2012-5	2.83	1.44	4.21	4.00	0.000
Mookiah et al., 2014-1	2.65	1.10	4.20	3.35	0.001
Mookiah et al., 2014-2	2.00	0.62	3.39	2.83	0.005
Mountzouris et al., 2015-1	-1.58	-2.78	-0.38	-2.58	0.010
Mountzouris et al., 2015-2	1.54	0.35	2.73	2.53	0.011
Mountzouris et al., 2007-1	1.84	0.36	3.32	2.44	0.015
Mountzouris et al., 2007-2	2.24	0.66	3.82	2.78	0.005
Mountzouris et al., 2010-1	-0.23	-1.28	0.83	-0.42	0.674
Mountzouris et al., 2010-2	0.08	-0.97	1.13	0.15	0.882
Mountzouris et al., 2010-3	0.91	-0.19	2.01	1.62	0.106
Mountzouris et al., 2010-4	-0.27	-1.32	0.79	-0.49	0.621
Mountzouris et al., 2010-5	0.58	-0.49	1.65	1.07	0.284
Mountzouris et al., 2010-6	2.55	1.14	3.96	3.55	0.000
Mountzouris et al., 2010-7	0.00	-1.05	1.05	0.00	1.000
Mountzouris et al., 2010-8	0.83	-0.27	1.92	1.48	0.138
Mountzouris et al., 2010-9	2.49	1.09	3.88	3.50	0.000
Rodriguez et al., 2012-1	1.70	1.00	2.41	4.73	0.000
Rodriguez et al., 2012-2	3.11	2.21	4.01	6.78	0.000
Sen et al., 2012-1	2.72	1.36	4.08	3.92	0.000
Sen et al., 2012-2	4.36	2.56	6.16	4.75	0.000
Sen et al., 2012-3	3.99	2.30	5.69	4.62	0.000
Sen et al., 2012-4	2.18	0.94	3.42	3.45	0.001
Sen et al., 2012-5	3.45	1.90	4.99	4.37	0.000
Sen et al., 2012-6	6.27	3.88	8.65	5.16	0.000
Shen et al., 2014-1	1.51	0.52	2.51	2.99	0.003
Shen et al., 2014-2	2.13	1.04	3.23	3.81	0.000
Shim et al., 2012-1	0.43	-0.56	1.42	0.85	0.397
Shim et al., 2012-2	1.02	-0.02	2.06	1.92	0.055
Shim et al., 2012-3	5.27	3.20	7.34	4.98	0.000
Shim et al., 2012-4	1.29	0.22	2.37	2.35	0.019
Yang et al., 2012-1	0.30	-0.84	1.43	0.51	0.611
Yang et al., 2012-2	0.28	-0.86	1.42	0.48	0.630
Yang et al., 2012-3	0.64	-0.52	1.80	1.08	0.279
Yang et al., 2012-4	-0.20	-1.33	0.94	-0.34	0.732
Yang et al., 2012-5	0.34	-0.80	1.48	0.59	0.554
Yang et al., 2012-6	0.36	-0.78	1.50	0.62	0.535
Yang et al., 2012-7	0.17	-0.97	1.30	0.29	0.771
Yang et al., 2012-8	0.22	-0.91	1.36	0.39	0.698
Yang et al., 2012-9	0.81	-0.37	1.99	1.35	0.177
Yang et al., 2012-10	0.00	-1.13	1.13	0.00	1.000
Yang et al., 2012-11	2.08	0.68	3.49	2.90	0.004
Yang et al., 2012-12	1.08	-0.13	2.29	1.75	0.080
Yang et al., 2012-13	0.43	-0.72	1.57	0.73	0.463
Yang et al., 2012-14	0.28	-0.86	1.41	0.48	0.632
Yang et al., 2012-15	0.90	-0.29	2.09	1.49	0.137
Yang et al., 2012-16	0.65	-0.51	1.81	1.10	0.270
Yang et al., 2012-17	2.80	1.21	4.40	3.45	0.001
Yang et al., 2012-18	2.32	0.86	3.79	3.11	0.002
	1.32	1.01	1.64	8.25	0.000

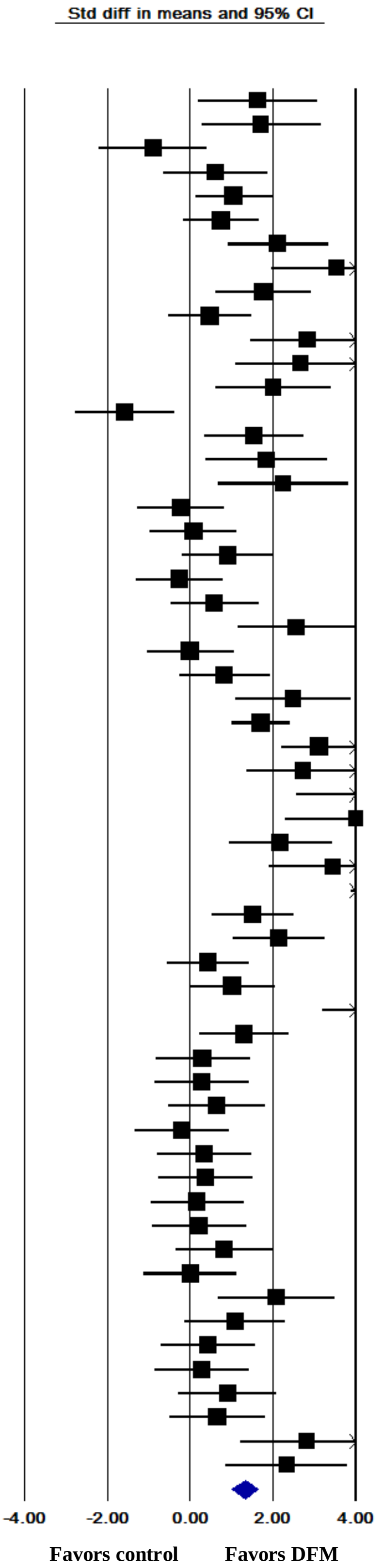

Figure S2. Forest plot for the effect of DFM supplementation on the log concentrations of *Bifidobacterium* in the gut of broiler chickens.

Study name	Std diff in means	Lower limit	Upper limit	Z-Value	p-Value
Yang et al., 2012-1	1.04	-0.17	2.25	1.69	0.091
Yang et al., 2012-2	4.04	2.07	6.02	4.01	0.000
Yang et al., 2012-3	1.86	0.51	3.22	2.69	0.007
Yang et al., 2012-4	8.33	4.81	11.84	4.64	0.000
Yang et al., 2012-5	3.74	1.87	5.62	3.91	0.000
Yang et al., 2012-6	14.79	8.76	20.81	4.81	0.000
Yang et al., 2012-7	9.06	5.26	12.86	4.68	0.000
	5.11	2.84	7.39	4.40	0.000

(Column group heading: Statistics for each study)

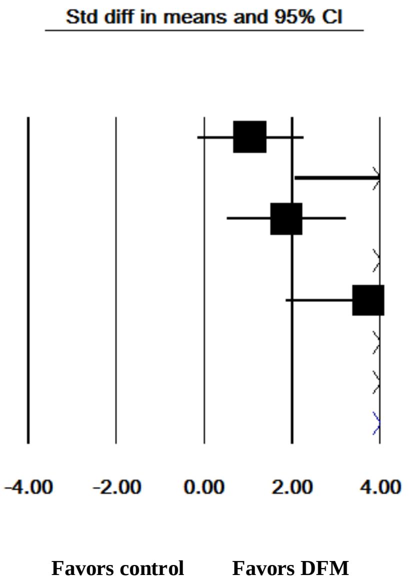

Figure S3. Forest plot for the effect of DFM supplementation on the log concentrations of *Clostridium butyricum* in the gut of broiler chickens.

Study name	Statistics for each study				
	Std diff in means	Lower limit	Upper limit	Z-Value	p-Value
Abudabos et al., 2015-1	-0.26	-1.24	0.73	-0.51	0.609
Boostani et al., 2013-1	-3.81	-5.71	-1.91	-3.93	0.000
Boostani et al., 2013-2	-5.54	-8.03	-3.05	-4.36	0.000
Gerard et al., 2008-1	-1.69	-2.83	-0.55	-2.90	0.004
Gerard et al., 2008-2	-0.25	-1.24	0.73	-0.51	0.612
Jeong and Kim, 2014-1	-0.29	-1.10	0.51	-0.71	0.477
Jeong and Kim, 2014-2	-0.36	-1.17	0.44	-0.89	0.376
Jeong and Kim, 2014-3	-0.27	-1.07	0.54	-0.65	0.517
Jeong and Kim, 2014-4	-0.73	-1.56	0.10	-1.73	0.083
Jeong and Kim, 2014-5	-1.33	-2.22	-0.45	-2.96	0.003
Jeong and Kim, 2014-6	-2.20	-3.21	-1.18	-4.25	0.000
Jeong and Kim, 2014-7	-0.46	-1.27	0.35	-1.11	0.265
Jeong and Kim, 2014-8	-0.92	-1.76	-0.08	-2.15	0.032
Kim et al., 2012-1	-4.38	-6.19	-2.58	-4.75	0.000
Kim et al., 2012-2	-4.36	-6.16	-2.56	-4.75	0.000
Kim et al., 2012-3	-5.23	-7.29	-3.17	-4.98	0.000
Kim et al., 2012-4	-5.30	-7.38	-3.22	-4.99	0.000
Kim et al., 2012-5	-5.09	-7.11	-3.07	-4.95	0.000
Kim et al., 2012-6	-5.77	-8.00	-3.54	-5.08	0.000
Mountzouris et al., 2015-1	-0.39	-1.45	0.66	-0.73	0.466
Mountzouris et al., 2015-2	2.79	1.32	4.26	3.71	0.000
Mountzouris et al., 2010-1	0.30	-0.76	1.35	0.55	0.580
Mountzouris et al., 2010-2	-1.23	-2.37	-0.09	-2.11	0.035
Mountzouris et al., 2010-3	0.58	-0.49	1.65	1.06	0.288
Mountzouris et al., 2010-4	0.38	-0.67	1.44	0.71	0.478
Mountzouris et al., 2010-5	-0.99	-2.10	0.12	-1.75	0.081
Mountzouris et al., 2010-6	0.18	-0.87	1.23	0.33	0.743
Mountzouris et al., 2010-7	0.00	-1.05	1.05	0.00	1.000
Mountzouris et al., 2010-8	-0.99	-2.10	0.12	-1.75	0.080
Mountzouris et al., 2010-9	-1.17	-2.31	-0.04	-2.03	0.042
Sen et al., 2012-1	-2.45	-3.75	-1.15	-3.70	0.000
Sen et al., 2012-2	-6.53	-8.99	-4.06	-5.19	0.000
Sen et al., 2012-3	-2.86	-4.25	-1.46	-4.02	0.000
Sen et al., 2012-4	-8.16	-11.15	-5.17	-5.34	0.000
Sen et al., 2012-5	-4.08	-5.80	-2.36	-4.65	0.000
Sen et al., 2012-6	-10.49	-14.25	-6.73	-5.46	0.000
Shim et al., 2012-1	-1.77	-2.92	-0.61	-3.00	0.003
Shim et al., 2012-2	-1.73	-2.88	-0.59	-2.96	0.003
Shim et al., 2012-3	-0.27	-1.26	0.71	-0.54	0.588
Shim et al., 2012-4	-2.70	-4.06	-1.35	-3.91	0.000
Yang et al., 2012-1	0.00	-1.13	1.13	0.00	1.000
Yang et al., 2012-2	-0.24	-1.38	0.89	-0.42	0.673
Yang et al., 2012-3	-0.44	-1.58	0.71	-0.75	0.453
Yang et al., 2012-4	-0.41	-1.55	0.74	-0.70	0.487
Yang et al., 2012-5	-0.82	-1.99	0.36	-1.36	0.175
Yang et al., 2012-6	-0.38	-1.52	0.76	-0.65	0.515
Yang et al., 2012-7	-0.14	-1.27	0.99	-0.24	0.809
Yang et al., 2012-8	-0.15	-1.28	0.98	-0.26	0.793
Yang et al., 2012-9	-2.58	-4.12	-1.05	-3.30	0.001
Yang et al., 2012-10	-0.93	-2.12	0.26	-1.53	0.126
Yang et al., 2012-11	-1.97	-3.35	-0.59	-2.80	0.005
Yang et al., 2012-12	-3.96	-5.91	-2.01	-3.99	0.000
Yang et al., 2012-13	-0.36	-1.51	0.78	-0.63	0.531
Yang et al., 2012-14	-0.71	-1.88	0.45	-1.20	0.231
Yang et al., 2012-15	-1.61	-2.91	-0.31	-2.42	0.015
Yang et al., 2012-16	-0.77	-1.94	0.40	-1.28	0.199
Yang et al., 2012-17	-3.93	-5.86	-1.99	-3.97	0.000
Yang et al., 2012-18	-4.08	-6.07	-2.10	-4.03	0.000
	-1.60	-2.00	-1.21	-8.00	0.000

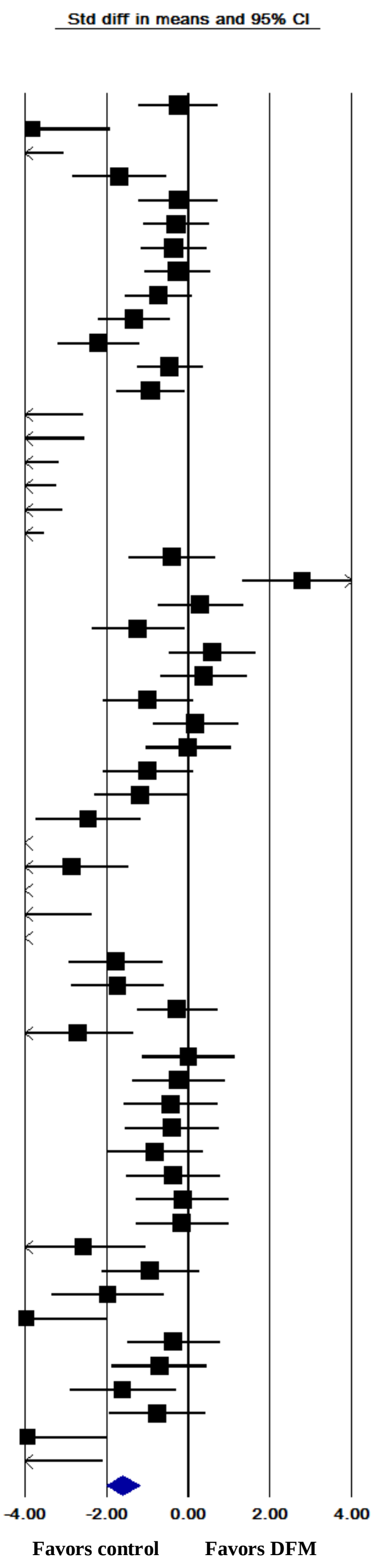

Figure S4. Forest plot for the effect of DFM supplementation on the log concentrations of *Clostridium perfringens* in the gut of broiler chickens.

Study name	Std diff in means	Lower limit	Upper limit	Z-Value	p-Value
Daskiran et al., 2012-1	-2.94	-4.94	-0.94	-2.88	0.004
Daskiran et al., 2012-2	1.50	0.39	2.61	2.65	0.008
Daskiran et al., 2012-3	-0.30	-1.29	0.68	-0.60	0.547
Deniz et al., 2011	-1.91	-2.64	-1.18	-5.13	0.000
Fajardo et al., 2012-1	-0.50	-1.65	0.65	-0.85	0.393
Fajardo et al., 2012-2	-0.48	-1.62	0.67	-0.81	0.417
Fajardo et al., 2012-3	-0.08	-1.21	1.05	-0.14	0.892
Fajardo et al., 2012-4	0.63	-0.53	1.79	1.07	0.286
Fajardo et al., 2012-5	0.81	-0.36	1.99	1.35	0.176
Fajardo et al., 2012-6	-0.71	-1.88	0.45	-1.20	0.232
Fajardo et al., 2012-7	0.77	-0.41	1.94	1.28	0.200
Fajardo et al., 2012-8	0.06	-1.07	1.19	0.11	0.915
Fajardo et al., 2012-9	0.51	-0.31	1.32	1.22	0.221
Fajardo et al., 2012-10	-0.23	-1.04	0.57	-0.57	0.568
Fajardo et al., 2012-11	-0.23	-1.03	0.57	-0.56	0.575
Fajardo et al., 2012-12	0.80	-0.03	1.64	1.89	0.058
Fajardo et al., 2012-13	0.16	-0.64	0.96	0.40	0.691
Fajardo et al., 2012-14	0.14	-0.66	0.94	0.35	0.727
Gerard et al., 2008-1	-0.14	-1.12	0.85	-0.27	0.786
Gerard et al., 2008-2	0.85	-0.17	1.87	1.63	0.103
Jin et al., 1998a-1	1.78	0.31	3.24	2.38	0.017
Jin et al., 1998a-2	-3.03	-4.85	-1.21	-3.27	0.001
Jin et al., 1998a-3	-1.91	-3.41	-0.42	-2.51	0.012
Jin et al., 1998a-4	-3.37	-5.29	-1.44	-3.42	0.001
Jin et al., 1998a-5	2.11	0.56	3.65	2.67	0.008
Jin et al., 1998a-6	-2.46	-4.10	-0.82	-2.93	0.003
Jin et al., 1998a-7	-1.50	-2.91	-0.10	-2.10	0.036
Jin et al., 1998a-8	-1.43	-2.82	-0.04	-2.02	0.044
Jin et al., 1998a-9	-1.46	-2.86	-0.07	-2.05	0.040
Jin et al., 1998a-10	2.42	0.79	4.05	2.91	0.004
Jin et al., 1998a-11	1.09	-0.23	2.42	1.61	0.106
Jin et al., 1998a-12	0.65	-0.62	1.92	1.00	0.316
Jin et al., 1998a-13	-1.93	-3.43	-0.43	-2.52	0.012
Jin et al., 1998a-14	-0.19	-1.43	1.06	-0.29	0.769
Jin et al., 1998a-15	-0.61	-1.88	0.66	-0.94	0.347
Jin et al., 1998a-16	-0.65	-1.92	0.62	-1.00	0.316
Jin et al., 1998b-1	-0.53	-1.43	0.36	-1.17	0.240
Jin et al., 1998b-2	-0.22	-1.10	0.66	-0.49	0.625
Jin et al., 1998b-3	0.15	-0.73	1.02	0.33	0.743
Jin et al., 1998b-4	-0.51	-1.40	0.38	-1.13	0.260
Jin et al., 1998b-5	-0.06	-0.94	0.82	-0.13	0.894
Jin et al., 1998b-6	-0.32	-1.20	0.56	-0.72	0.474
Jin et al., 1998b-7	0.09	-0.79	0.96	0.20	0.844
Jin et al., 1998b-8	0.15	-0.73	1.03	0.34	0.737
Jin et al., 1998b-9	-0.20	-1.08	0.67	-0.46	0.648
Jin et al., 1998b-10	-0.33	-1.21	0.55	-0.73	0.465
Jin et al., 1998b-11	0.30	-0.58	1.18	0.67	0.500
Jin et al., 1998b-12	-0.24	-1.12	0.64	-0.53	0.595
Jin et al., 1998b-13	-1.64	-2.66	-0.63	-3.18	0.001
Jin et al., 1998b-14	-1.12	-2.07	-0.18	-2.34	0.019
Jin et al., 1998b-15	-0.60	-1.50	0.29	-1.32	0.188
Jin et al., 1998b-16	0.14	-0.73	1.02	0.32	0.748
Jin et al., 1998b-17	-1.37	-2.34	-0.40	-2.76	0.006
Jin et al., 1998b-18	-0.97	-1.89	-0.04	-2.04	0.041
Jin et al., 1998b-19	0.31	-0.58	1.19	0.68	0.497
Jin et al., 1998b-20	0.37	-0.51	1.26	0.83	0.406
Jin et al., 1998b-21	-1.25	-2.21	-0.29	-2.55	0.011
Jin et al., 1998b-22	-0.53	-1.43	0.36	-1.17	0.241
Jin et al., 1998b-23	0.02	-0.86	0.89	0.04	0.968
Jin et al., 1998b-24	0.14	-0.73	1.02	0.32	0.748
Kim et al., 2012-1	-4.71	-6.62	-2.81	-4.85	0.000
Kim et al., 2012-2	-3.65	-5.25	-2.05	-4.47	0.000
Kim et al., 2012-3	-5.18	-7.23	-3.14	-4.97	0.000
Kim et al., 2012-4	-5.18	-7.23	-3.14	-4.97	0.000
Kim et al., 2012-5	-5.30	-7.38	-3.22	-4.99	0.000
Kim et al., 2012-6	-5.06	-7.07	-3.05	-4.94	0.000
Liu et al., 2007-1	-1.79	-3.44	-0.15	-2.14	0.032
Liu et al., 2007-2	-6.21	-9.55	-2.86	-3.64	0.000
Liu et al., 2007-3	-1.35	-2.89	0.18	-1.73	0.084
Liu et al., 2007-4	2.04	0.33	3.75	2.34	0.019
Milián et al., 2013-1	-0.04	-0.92	0.84	-0.09	0.928
Milián et al., 2013-2	-1.11	-2.05	-0.17	-2.30	0.021
Milián et al., 2013-3	-0.04	-0.92	0.84	-0.09	0.928
Milián et al., 2013-4	-1.11	-2.05	-0.17	-2.30	0.021
Milián et al., 2013-5	-0.12	-1.00	0.76	-0.27	0.788
Milián et al., 2013-6	-1.11	-2.05	-0.17	-2.30	0.021
Mountzouris et al., 2007-1	-0.09	-1.33	1.15	-0.15	0.884
Mountzouris et al., 2007-2	-0.19	-1.43	1.06	-0.30	0.768
Mountzouris et al., 2010-1	0.35	-0.71	1.40	0.65	0.517
Mountzouris et al., 2010-2	-0.69	-1.77	0.39	-1.26	0.209
Mountzouris et al., 2010-3	0.05	-0.99	1.10	0.10	0.919
Mountzouris et al., 2010-4	-1.18	-2.32	-0.05	-2.04	0.041
Mountzouris et al., 2010-5	-0.29	-1.34	0.76	-0.54	0.590
Mountzouris et al., 2010-6	2.46	1.07	3.84	3.47	0.001
Mountzouris et al., 2010-7	0.62	-0.46	1.69	1.12	0.261
Mountzouris et al., 2010-8	-0.32	-1.38	0.73	-0.60	0.548
Mountzouris et al., 2010-9	-1.85	-3.10	-0.59	-2.89	0.004
Rada and Marounek, 1997-1	-2.26	-3.84	-0.67	-2.79	0.005
Rada and Marounek, 1997-2	0.86	-0.44	2.16	1.30	0.193
Rada and Marounek, 1997-3	-2.24	-3.83	-0.66	-2.78	0.005
Rada and Marounek, 1997-4	0.46	-0.80	1.71	0.71	0.481
Sen et al., 2012-1	-1.31	-2.39	-0.23	-2.37	0.018
Sen et al., 2012-2	-8.99	-12.26	-5.73	-5.40	0.000
Sen et al., 2012-3	-1.74	-2.89	-0.59	-2.97	0.003
Sen et al., 2012-4	-10.35	-14.07	-6.64	-5.46	0.000
Sen et al., 2012-5	-1.20	-2.26	-0.13	-2.20	0.027
Sen et al., 2012-6	-9.26	-12.62	-5.91	-5.41	0.000
Shim et al., 2012-1	-2.35	-3.62	-1.07	-3.61	0.000
Shim et al., 2012-2	-2.50	-3.80	-1.19	-3.74	0.000
Shim et al., 2012-3	-1.89	-3.07	-0.71	-3.14	0.002
Shim et al., 2012-4	-1.84	-3.01	-0.67	-3.08	0.002
Yakhkeshi et al., 2012-1	-3.55	-4.95	-2.14	-4.94	0.000
Yakhkeshi et al., 2012-2	-2.80	-4.03	-1.57	-4.45	0.000
Yakhkeshi et al., 2012-3	-1.13	-2.08	-0.19	-2.35	0.019
Zhang et al., 2016-1	-2.33	-3.46	-1.19	-4.02	0.000
Zhang et al., 2016-2	-0.40	-1.29	0.48	-0.89	0.371
	-0.81	-1.05	-0.56	-6.41	0.000

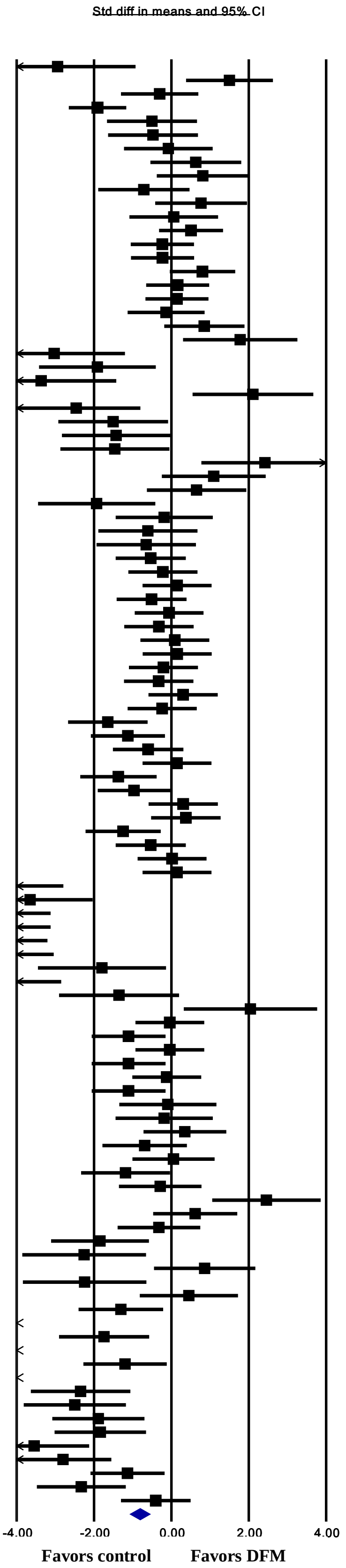

Figure S5. Forest plot for the effect of DFM supplementation on the log concentrations of coliforms in the gut of broiler chickens.

Study name	Std diff in means	Lower limit	Upper limit	Z-Value	p-Value
Ahmed et al., 2014-1	-0.25	-0.77	0.28	-0.92	0.355
Ahmed et al., 2014-2	-0.83	-1.38	-0.29	-2.99	0.003
Ahmed et al., 2014-3	-1.11	-1.67	-0.55	-3.86	0.000
Ahmed et al., 2014-4	-1.85	-2.48	-1.23	-5.80	0.000
Altaher et al., 2015	-0.80	-1.63	0.03	-1.89	0.059
Jeong and Kim, 2014-1	-0.34	-1.15	0.46	-0.83	0.404
Jeong and Kim, 2014-2	-0.91	-1.75	-0.07	-2.12	0.034
Jeong and Kim, 2014-3	-2.13	-3.13	-1.12	-4.16	0.000
Jeong and Kim, 2014-4	-3.86	-5.21	-2.51	-5.59	0.000
Jeong and Kim, 2014-5	-0.24	-1.04	0.57	-0.58	0.564
Jeong and Kim, 2014-6	-0.73	-1.55	0.10	-1.72	0.085
Jeong and Kim, 2014-7	-1.80	-2.75	-0.85	-3.72	0.000
Jeong and Kim, 2014-8	-1.96	-2.94	-0.99	-3.95	0.000
Jin et al., 1996-1	-0.18	-1.78	1.42	-0.22	0.826
Jin et al., 1996-2	-1.49	-3.30	0.32	-1.62	0.106
Jin et al., 1996-3	-0.52	-2.15	1.10	-0.63	0.528
Jin et al., 1996-4	-0.95	-2.64	0.74	-1.10	0.271
Jin et al., 1996-5	-0.52	-2.15	1.11	-0.63	0.531
Jin et al., 1996-6	-1.74	-3.61	0.14	-1.81	0.070
Jin et al., 1996-7	-1.97	-3.92	-0.02	-1.98	0.048
Jin et al., 1996-8	-0.93	-2.62	0.75	-1.08	0.278
Jin et al., 1996-9	-0.56	-2.19	1.07	-0.67	0.503
Jin et al., 1996-10	-2.41	-4.52	-0.31	-2.25	0.025
Jin et al., 1996-11	-1.02	-2.72	0.68	-1.18	0.240
Jin et al., 1996-12	-2.27	-4.32	-0.22	-2.17	0.030
Lei et al., 2015-1	0.00	-1.12	1.12	0.00	1.000
Lei et al., 2015-2	0.00	-1.12	1.12	0.00	1.000
Lei et al., 2015-3	-0.06	-1.44	1.33	-0.08	0.935
Lei et al., 2015-4	-0.92	-2.38	0.53	-1.24	0.214
Lei et al., 2015-5	-1.10	-2.58	0.39	-1.45	0.148
Lei et al., 2015-6	-2.76	-4.69	-0.82	-2.79	0.005
Lei et al., 2015-7	-2.76	-4.69	-0.82	-2.79	0.005
Lei et al., 2015-8	-2.83	-4.80	-0.87	-2.83	0.005
Mohammadi Gheisar et al., 2016-1	-0.69	-1.59	0.22	-1.49	0.136
Mohammadi Gheisar et al., 2016-2	-0.53	-1.42	0.36	-1.16	0.247
Mookiah et al., 2014-1	-3.02	-4.67	-1.36	-3.57	0.000
Mookiah et al., 2014-2	-1.04	-2.24	0.17	-1.69	0.091
Mountzouris et al., 2015-1	-0.15	-1.20	0.89	-0.29	0.772
Mountzouris et al., 2015-2	1.45	0.27	2.63	2.42	0.016
Peng et al., 2016-1	-2.05	-3.45	-0.65	-2.88	0.004
Peng et al., 2016-2	-2.96	-4.59	-1.32	-3.54	0.000
Peng et al., 2016-3	-0.58	-1.73	0.58	-0.98	0.327
Peng et al., 2016-4	-3.70	-5.57	-1.84	-3.89	0.000
Rodriguez et al., 2012-1	0.43	-0.18	1.04	1.37	0.171
Rodriguez et al., 2012-2	-1.09	-1.74	-0.44	-3.30	0.001
Salim et al., 2013-1	-1.92	-3.10	-0.73	-3.17	0.002
Salim et al., 2013-2	-2.84	-4.22	-1.45	-4.01	0.000
Shen et al., 2014-1	-1.53	-2.52	-0.53	-3.01	0.003
Shen et al., 2014-2	-2.02	-3.10	-0.95	-3.68	0.000
Wu et al., 2011-1	-10.68	-16.93	-4.43	-3.35	0.001
Wu et al., 2011-2	-8.90	-14.18	-3.62	-3.30	0.001
Wu et al., 2011-3	-0.80	-2.47	0.86	-0.95	0.344
Wu et al., 2011-4	-6.28	-10.17	-2.38	-3.16	0.002
Wu et al., 2011-5	-8.20	-13.11	-3.29	-3.27	0.001
Wu et al., 2011-6	-7.88	-12.62	-3.14	-3.26	0.001
Wu et al., 2011-7	-2.26	-4.31	-0.21	-2.16	0.030
Wu et al., 2011-8	-11.64	-18.42	-4.86	-3.37	0.001
Wu et al., 2011-9	-0.94	-2.63	0.75	-1.09	0.274
Wu et al., 2011-10	-11.70	-18.51	-4.89	-3.37	0.001
Wu et al., 2011-11	0.33	-1.28	1.94	0.40	0.686
Wu et al., 2011-12	-15.29	-24.09	-6.49	-3.41	0.001
Yang et al., 2012-1	-0.11	-1.24	1.03	-0.19	0.853
Yang et al., 2012-2	-0.22	-1.35	0.92	-0.37	0.708
Yang et al., 2012-3	-0.09	-1.22	1.05	-0.15	0.881
Yang et al., 2012-4	-1.64	-2.94	-0.33	-2.45	0.014
Yang et al., 2012-5	0.00	-1.13	1.13	0.00	1.000
Yang et al., 2012-6	-0.67	-1.84	0.49	-1.13	0.256
Yang et al., 2012-7	-0.20	-1.33	0.94	-0.34	0.735
Yang et al., 2012-8	-0.61	-1.77	0.55	-1.04	0.300
Yang et al., 2012-9	-0.84	-2.02	0.34	-1.40	0.162
Yang et al., 2012-10	-4.97	-7.25	-2.68	-4.26	0.000
Yang et al., 2012-11	0.15	-0.98	1.29	0.27	0.791
Yang et al., 2012-12	-1.68	-3.00	-0.37	-2.50	0.012
Yang et al., 2012-13	-0.22	-1.36	0.91	-0.38	0.703
Yang et al., 2012-14	-0.43	-1.58	0.71	-0.74	0.457
Yang et al., 2012-15	-0.13	-1.26	1.00	-0.22	0.824
Yang et al., 2012-16	-5.90	-8.51	-3.28	-4.42	0.000
Yang et al., 2012-17	-0.24	-1.38	0.89	-0.42	0.677
Yang et al., 2012-18	-2.45	-3.94	-0.95	-3.20	0.001
Zhang et al., 2013-1	-1.17	-1.87	-0.46	-3.23	0.001
Zhang et al., 2013-2	0.66	-0.01	1.33	1.93	0.053
Zhang and Kim, 2014-1	-1.36	-2.16	-0.57	-3.36	0.001
Zhang and Kim, 2014-2	-1.44	-2.24	-0.63	-3.50	0.000
	-1.20	-1.45	-0.94	-9.17	0.000

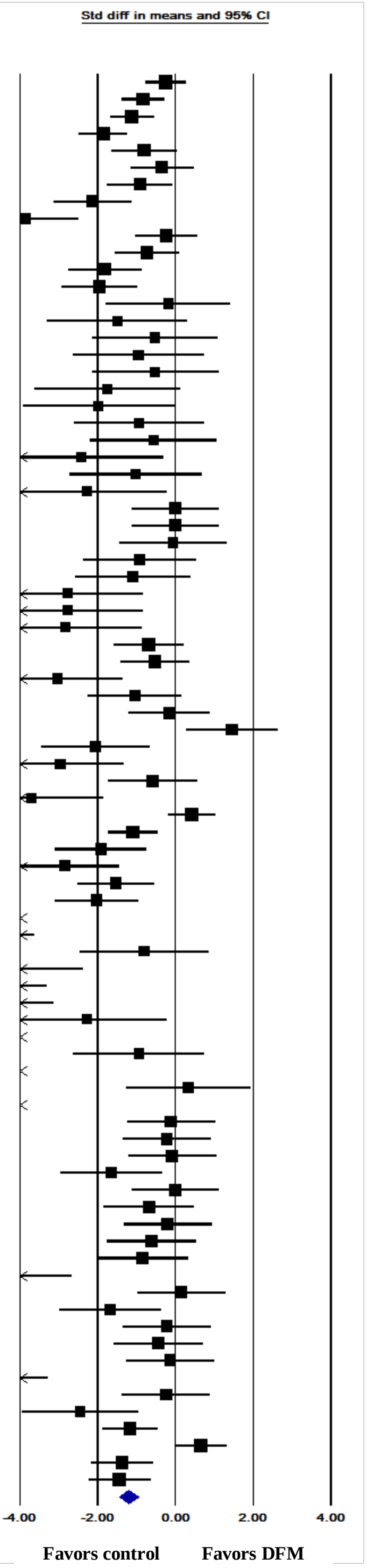

Figure S6. Forest plot for the effect of DFM supplementation on the log concentrations of *Escherichia coli* in the gut of broiler chickens.

Study name	Std diff in means	Lower limit	Upper limit	Z-Value	p-Value
Altaher et al., 2015	-0.16	-0.96	0.64	-0.39	0.696
Deniz et al., 2011	-3.11	-4.01	-2.22	-6.79	0.000
Rada and Marounek, 1997-1	-2.47	-4.12	-0.83	-2.94	0.003
Rada and Marounek, 1997-2	-0.91	-2.22	0.39	-1.38	0.169
Rada and Marounek, 1997-3	-1.84	-3.31	-0.36	-2.43	0.015
Rada and Marounek, 1997-4	-0.51	-1.77	0.75	-0.80	0.423
Shen et al., 2014-1	-0.26	-1.14	0.62	-0.58	0.565
Shen et al., 2014-2	-0.80	-1.71	0.11	-1.72	0.085
	-1.21	-2.04	-0.38	-2.86	0.004

Statistics for each study

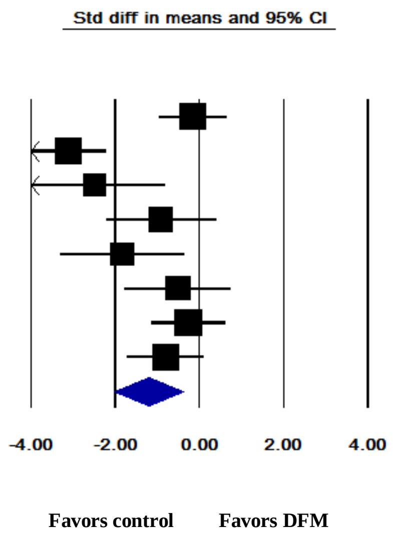

Figure S7. Forest plot for the effect of DFM supplementation on the log concentrations of *Enterococcus* in the gut of broiler chickens.

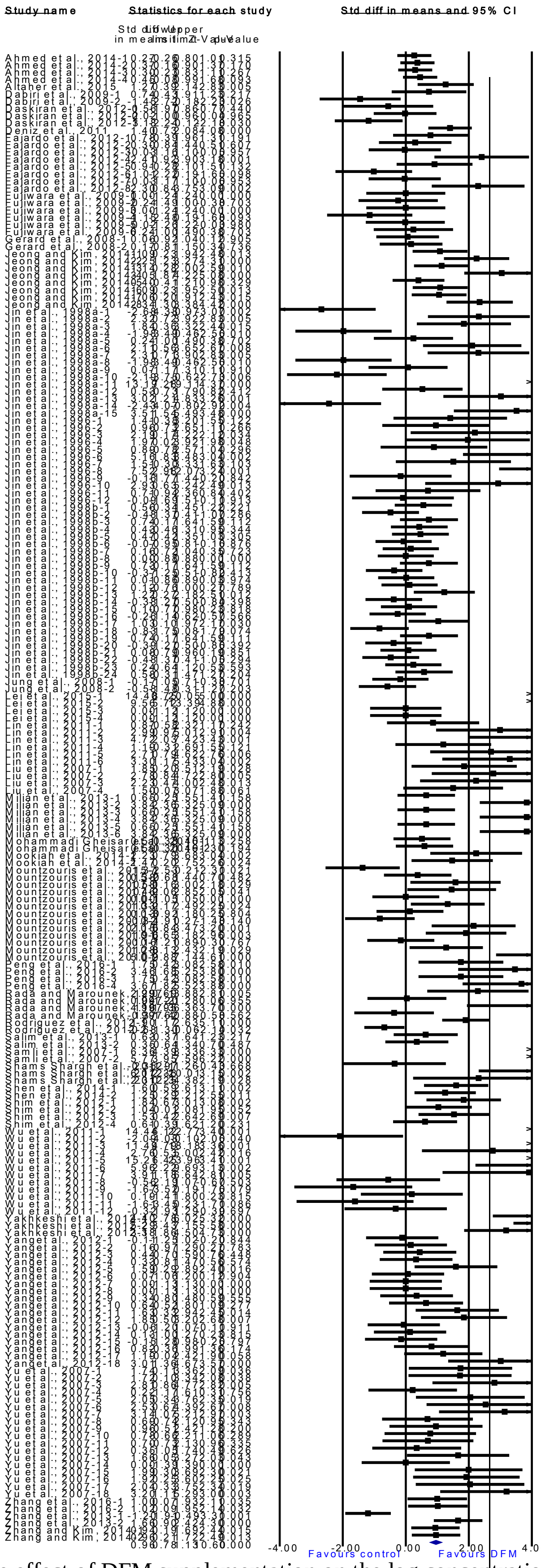

Figure S8. A forest plot for the effect of DFM supplementation on the log concentrations of *Lactobacillus* in the gut of broiler chickens.

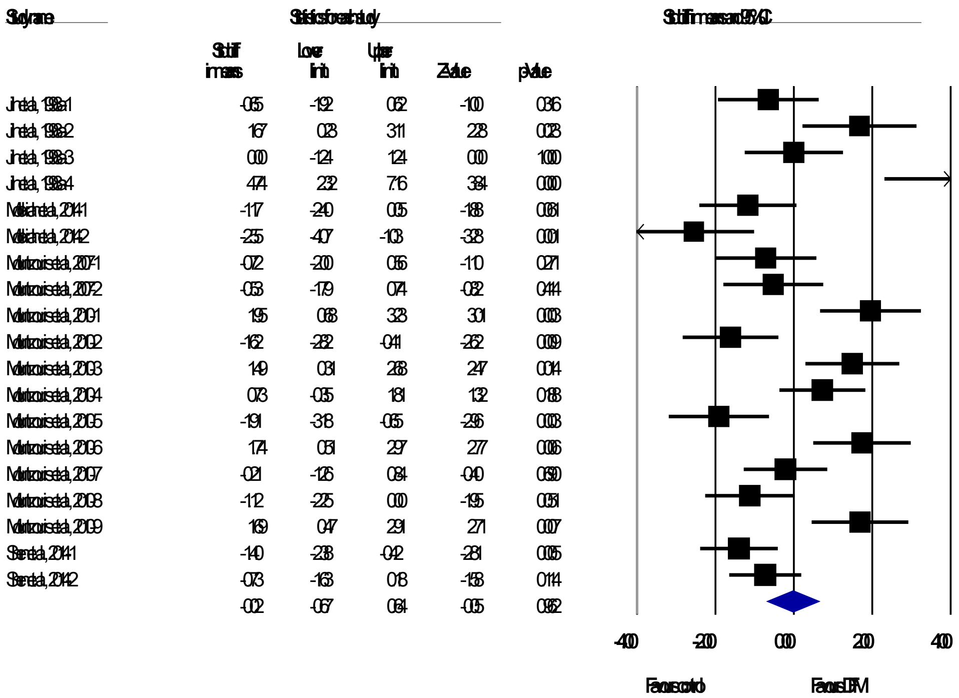

Figure S9. Forest plot for the effect of DFM supplementation on the log concentrations of aerobes in the gut of broiler chickens.

Study name	Std diff in means	Lower limit	Upper limit	Z-Value	p-Value
	Statistics for each study				
Daskiran et al., 2012-1	-1.71	-3.34	-0.09	-2.07	0.038
Daskiran et al., 2012-2	1.09	0.04	2.14	2.03	0.042
Daskiran et al., 2012-3	-0.72	-1.73	0.29	-1.40	0.162
Fujiwara et al., 2009-1	0.58	-0.68	1.85	0.91	0.365
Fujiwara et al., 2009-2	-0.15	-1.39	1.10	-0.23	0.817
Fujiwara et al., 2009-3	-0.44	-1.69	0.82	-0.69	0.493
Fujiwara et al., 2009-4	-0.44	-1.69	0.82	-0.69	0.493
Fujiwara et al., 2009-5	2.05	0.52	3.58	2.62	0.009
Fujiwara et al., 2009-6	0.88	-0.42	2.17	1.32	0.185
Jin et al., 1998a-1	2.80	1.05	4.54	3.14	0.002
Jin et al., 1998a-2	0.65	-0.62	1.92	1.00	0.318
Jin et al., 1998a-3	-12.70	-18.40	-7.00	-4.37	0.000
Jin et al., 1998a-4	-11.90	-17.27	-6.54	-4.35	0.000
Jin et al., 1996-1	0.21	-1.40	1.81	0.25	0.801
Jin et al., 1996-2	-0.62	-2.26	1.02	-0.74	0.460
Jin et al., 1996-3	-0.21	-1.82	1.39	-0.26	0.793
Jin et al., 1996-4	-0.15	-1.76	1.45	-0.19	0.851
Jin et al., 1996-5	1.02	-0.68	2.72	1.17	0.241
Jin et al., 1996-6	-0.07	-1.67	1.53	-0.09	0.930
Jin et al., 1996-7	0.44	-1.18	2.06	0.54	0.592
Jin et al., 1996-8	0.33	-1.28	1.94	0.40	0.689
Jin et al., 1996-9	-0.22	-1.82	1.39	-0.27	0.791
Jung et al., 2008-1	1.18	0.23	2.13	2.43	0.015
Jung et al., 2008-2	-0.33	-1.21	0.55	-0.73	0.465
Kim et al., 2012-1	-0.47	-1.47	0.52	-0.93	0.352
Kim et al., 2012-2	-1.41	-2.51	-0.32	-2.53	0.011
Kim et al., 2012-3	-0.71	-1.72	0.30	-1.37	0.170
Kim et al., 2012-4	0.00	-0.98	0.98	0.00	1.000
Kim et al., 2012-5	-0.47	-1.47	0.52	-0.93	0.352
Kim et al., 2012-6	1.06	0.01	2.11	1.99	0.047
Liu et al., 2007-1	6.29	2.91	9.67	3.65	0.000
Liu et al., 2007-2	1.65	0.05	3.26	2.02	0.043
Liu et al., 2007-3	0.66	-0.77	2.08	0.90	0.366
Liu et al., 2007-4	0.34	-1.06	1.74	0.48	0.633
Milián et al., 2013-1	0.59	-0.31	1.48	1.28	0.200
Milián et al., 2013-2	1.58	0.58	2.58	3.09	0.002
Milián et al., 2013-3	0.59	-0.31	1.48	1.28	0.200
Milián et al., 2013-4	1.58	0.58	2.58	3.09	0.002
Milián et al., 2013-5	0.59	-0.31	1.48	1.28	0.200
Milián et al., 2013-6	1.58	0.58	2.58	3.09	0.002
Mountzouris et al., 2007-1	-0.60	-1.87	0.66	-0.93	0.351
Mountzouris et al., 2007-2	-0.34	-1.59	0.90	-0.54	0.588
Mountzouris et al., 2010-1	-2.08	-3.37	-0.78	-3.13	0.002
Mountzouris et al., 2010-2	-2.57	-3.98	-1.15	-3.56	0.000
Mountzouris et al., 2010-3	-1.10	-2.22	0.02	-1.92	0.055
Mountzouris et al., 2010-4	1.19	0.06	2.33	2.06	0.040
Mountzouris et al., 2010-5	-3.30	-4.91	-1.69	-4.02	0.000
Mountzouris et al., 2010-6	0.42	-0.64	1.48	0.78	0.436
Mountzouris et al., 2010-7	-0.18	-1.23	0.87	-0.34	0.733
Mountzouris et al., 2010-8	-2.72	-4.17	-1.27	-3.67	0.000
Mountzouris et al., 2010-9	-0.75	-1.83	0.33	-1.36	0.175
Sen et al., 2012-1	-3.80	-5.45	-2.16	-4.54	0.000
Sen et al., 2012-2	1.63	0.50	2.76	2.83	0.005
Sen et al., 2012-3	-1.09	-2.14	-0.04	-2.03	0.042
Sen et al., 2012-4	-2.04	-3.25	-0.83	-3.31	0.001
Sen et al., 2012-5	-4.35	-6.14	-2.55	-4.74	0.000
Sen et al., 2012-6	-8.57	-11.70	-5.44	-5.37	0.000
Shen et al., 2014-1	1.63	0.62	2.64	3.15	0.002
Shen et al., 2014-2	0.47	-0.41	1.36	1.05	0.296
Shim et al., 2012-1	-1.48	-2.59	-0.37	-2.62	0.009
Shim et al., 2012-2	-0.73	-1.74	0.28	-1.41	0.159
Shim et al., 2012-3	0.31	-0.68	1.29	0.61	0.543
Shim et al., 2012-4	-1.22	-2.29	-0.16	-2.25	0.025
Yakhkeshi et al., 2012-1	-2.73	-3.95	-1.51	-4.39	0.000
Yakhkeshi et al., 2012-2	-1.90	-2.96	-0.85	-3.53	0.000
Yakhkeshi et al., 2012-3	-1.86	-2.91	-0.81	-3.48	0.001
	-0.34	-0.70	0.02	-1.84	0.066

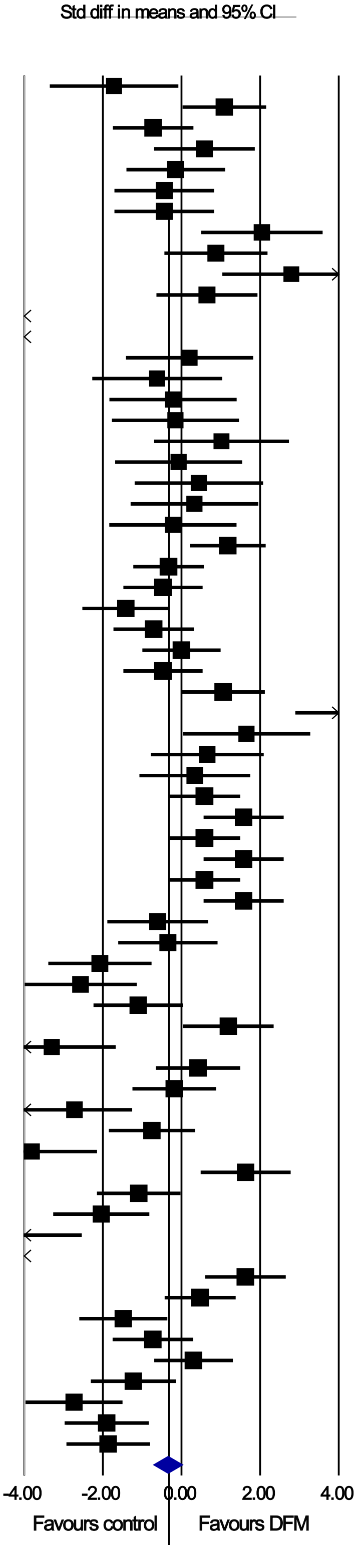

Figure S10. Forest plot for the effect of DFM supplementation on the log concentrations of anaerobes in the gut of broiler chickens.

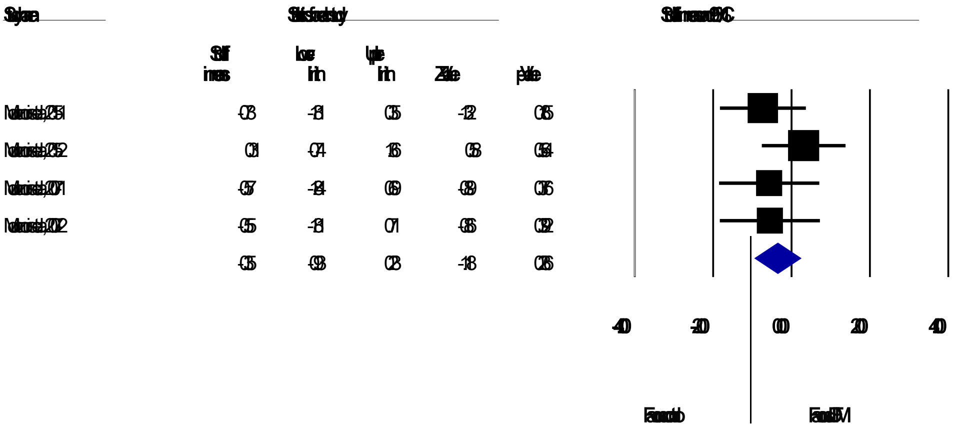

Figure S11. Forest plot for the effect of DFM supplementation on the log concentrations of *Bacteroides* in the gut of broiler chickens.

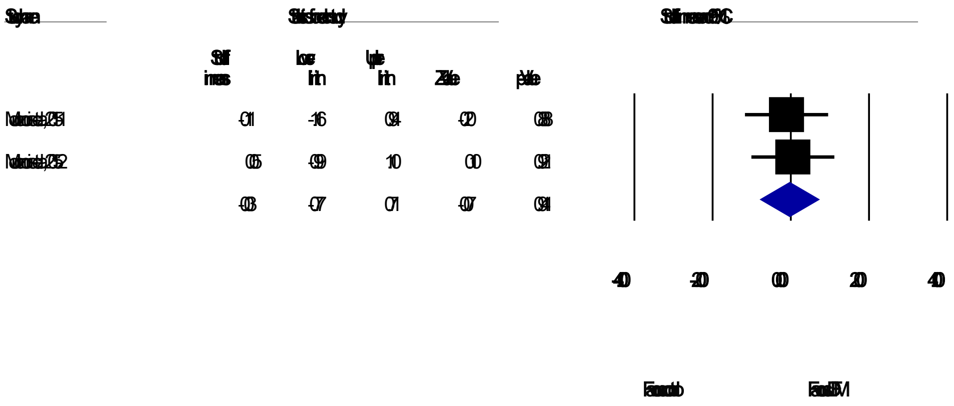

Figure S12. Forest plot for the effect of DFM supplementation on the log concentrations of *Clostridium coccoides* in the gut of broiler chickens.

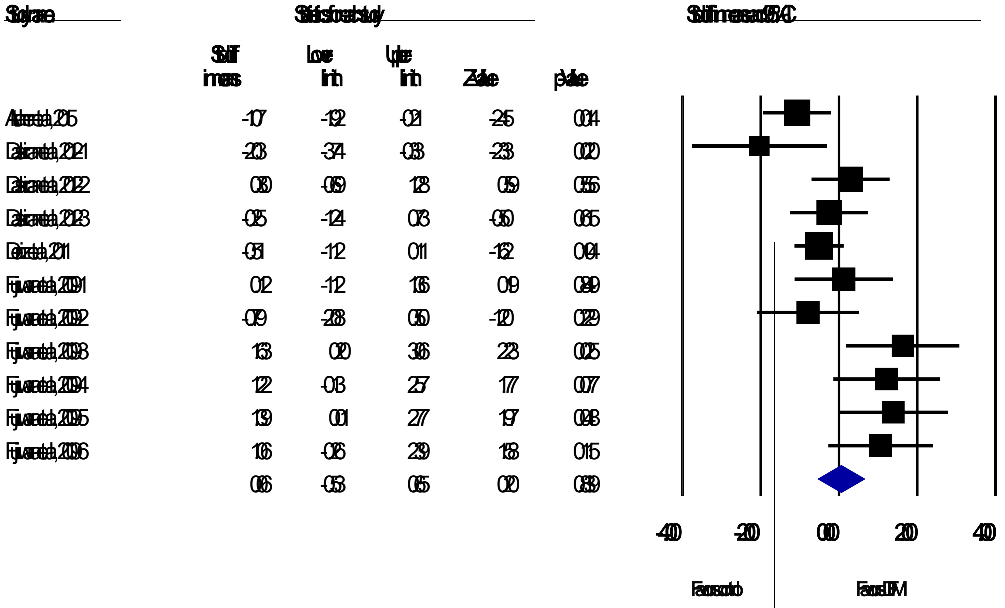

Figure S13. Forest plot for the effect of DFM supplementation on the concentrations of *Enterobateriaceae* in the gut of broiler chickens.

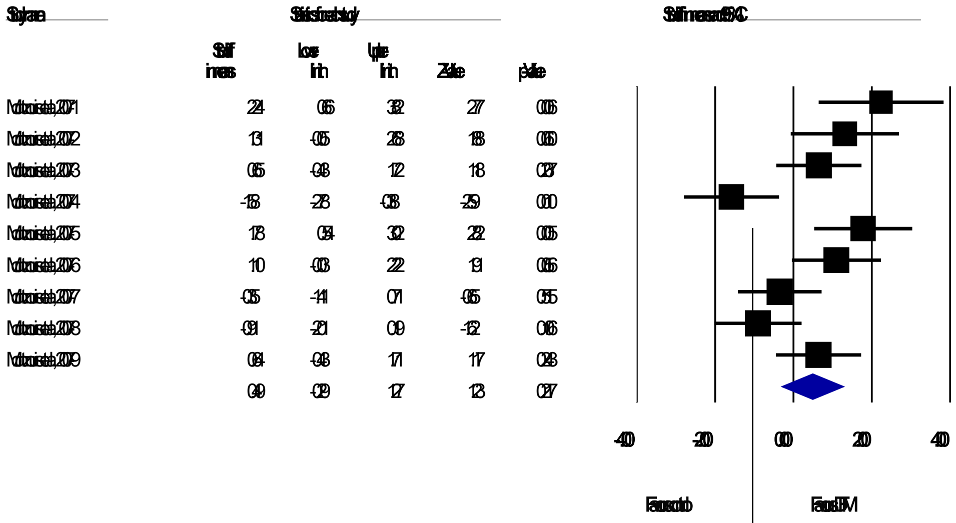

Figure S14. Forest plot for the effect of DFM supplementation on the log concentrations of Gram positive cocci in the gut of broiler chickens.

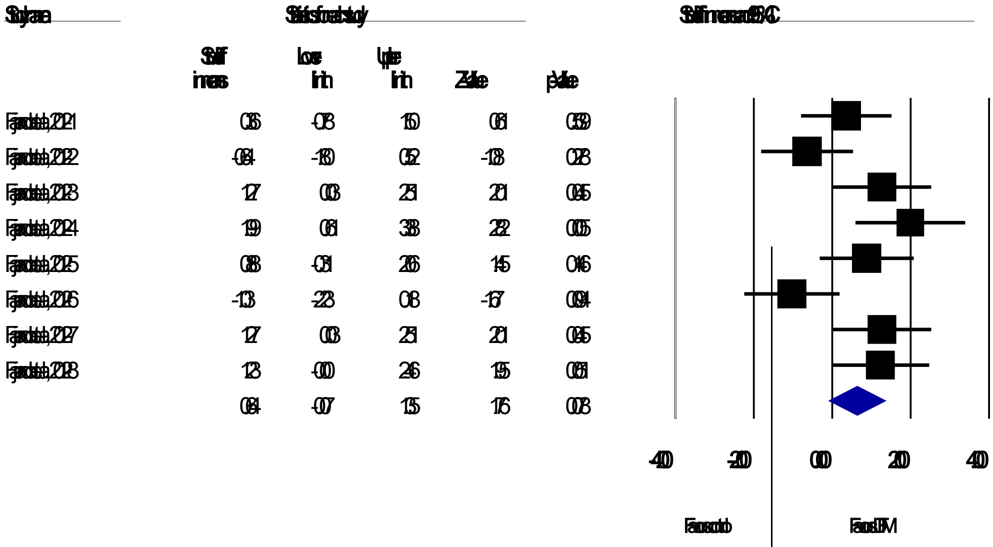

Figure S15. Forest plot for the effect of DFM supplementation on the log concentrations of mesophilic bacteria in the gut of broiler chickens.

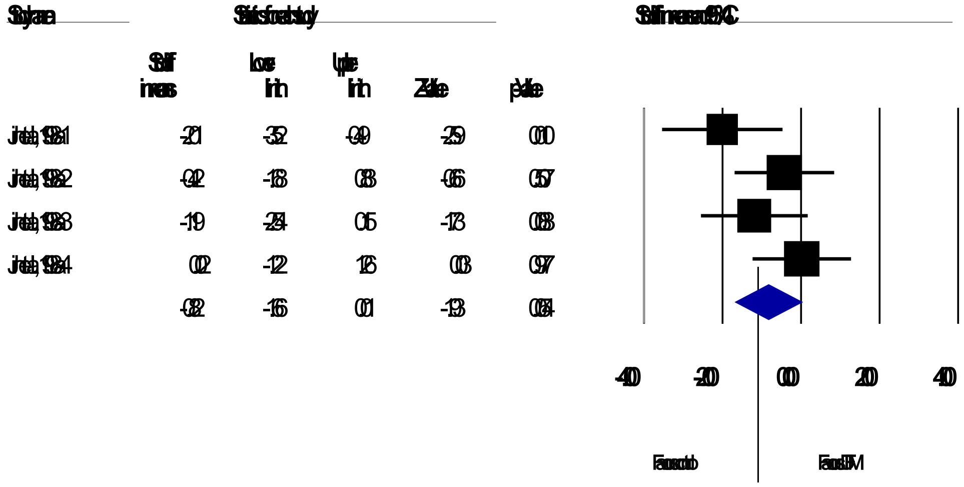

Figure S16. Forest plot for the effect of DFM supplementation on the log concentrations of *Streptococcus* in the gut of broiler chickens.

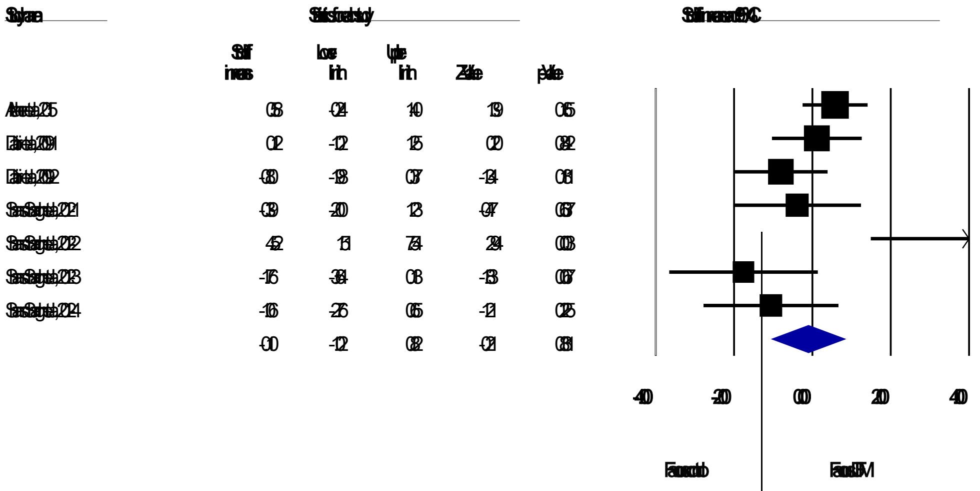

Figure S17. Forest plot for the effect of DFM supplementation on the log concentrations of total bacterial counts in the gut of broiler chickens.
